# Autoantibody against β_1_-adrenoceptor promotes the differentiation of natural regulatory T cells from activated CD4^+^ T cells by up-regulating AMPK-mediated fatty acid oxidation

**DOI:** 10.1038/s41419-018-1209-2

**Published:** 2019-02-15

**Authors:** Wenli Xu, Ye Wu, Li Wang, Yan Bai, Yunhui Du, Yang Li, Ning Cao, Yuhui Zhao, Youyi Zhang, Huirong Liu

**Affiliations:** 1 Department of Physiology and Pathophysiology, School of Basic Medical Sciences, Capital Medical University, 100069 Beijing, China; 2 Department of Pathology, School of Basic Medical Sciences, Shanxi Medical University, 030001 Taiyuan, China; 3 Beijing Anzhen Hospital, Capital Medical University, 100029 Beijing, China; 4 Department of Cardiology and Institute of Vascular Medicine, Peking University Third Hospital, 100191 Beijing, China; 5 Beijing Key Laboratory of Cardiovascular Receptors Research, 100191 Beijing, China; 6 Beijing Key Laboratory of Cardiovascular Diseases Related to Metabolic Disturbance, 100069 Beijing, China

## Abstract

Therapeutic adoptive transfer of natural regulatory T cells (nTreg, CD4^+^ CD25^+^ Foxp3^+^ T cells) or in vivo selective expansion of nTreg cells has been demonstrated to improve the cardiac function in various cardiovascular disease models. The differentiation of nTreg cells is mediated by catecholamines via β_1_-adrenergic receptor (β_1_-AR) activation. Autoantibody against β_1_-adrenoceptor (β_1_-AA) as a β_1_-AR agonist is closely associated with the occurrence and deterioration of cardiac dysfunction. However, whether β_1_-AA has any impact on nTreg cells has not been reported. The aim of the present study was intended to assess the potential impact of β_1_-AA on nTreg cell differentiation and explore the underlying mechanism. It was found that the expression of multiple proteins involved in nTreg cell differentiation, immunosuppressive function, and migration was up-regulated in mice after β_1_-AA administration, suggesting that β_1_-AA may promote nTreg cell activation. In vitro, β_1_-AA promoted nTreg cell differentiation by up-regulating mitochondrial fatty acid oxidation (FAO) in activated CD4^+^ T cells via AMP-activated protein kinase (AMPK) activation and mitochondrial membrane potential reduction. In addition, the AMPK agonist facilitated β_1_-AA-mediated FAO and nTreg cell differentiation. To further confirm the role of AMPK in β_1_-AA-mediated nTreg cell differentiation, β_1_-AA was acted on the CD4^+^ T cells isolated from AMPK-deficient (AMPK^−/−^) mice. The result showed that the effect of β_1_-AA on nTreg cell differentiation was attenuated markedly after AMPK knockout. In conclusion, AMPK-mediated metabolic regulation targeting for nTreg cell restoration may be a promising therapeutic target for β_1_-AA-positive patients with cardiac dysfunction.

## Introduction

CD4^+^ T cells are known as the most important participant in adaptive immunity of the organism. Over-activation of CD4^+^ T cells and disproportion of their subpopulations play an important role in the pathogenesis of various cardiovascular diseases. Functionally, CD4^+^ T cells are classified as two major categories: effector T cells and regulatory T (Treg) cells^1^, among which natural Treg (nTreg, CD4^+^ CD25^+^ Foxp3^+^ T) cells play a critical role in inhibiting the immune response of effector T cells and maintaining immune tolerance^2,3^. Therapeutic adoptive transfer of nTreg cells or in vivo selective nTreg cell expansion has been demonstrated to attenuate post-infraction left ventricular remodeling, relief myocardial injury, and eventually improve the cardiac function in diverse cardiovascular disease models^4,5^. Studies have confirmed that the development and function of nTreg cells are regulated by catecholamines via the expression of α-, β_1_-, and β_2_-adrenergic receptors (β_1/2_-ARs)^6–8^. Compared with effector T cells, β_1_-AR expression in nTreg cells is more advantageous than β_2_-AR expression^8^, but the effect of β_1_-AR activation on nTreg cells remains unclear.

Autoantibody targeting the second extracellular loop of β_1_-adrenoceptor (β_1_-AA) is commonly detected in circulating blood of the patients with cardiac dysfunction caused by etiologies like dilated cardiomyopathy, ischemic heart disease, and arrhythmia^9–11^. β_1_-AA was found to exhibit the agonist-like effects on β_1_-AR, such as increasing the intracellular calcium level promoting the beating frequency of neonatal rat cardiomyocytes and inducing cAMP production^12–14^. The positive rate of β_1_-AA was reported to be as high as 80% in different cardiac dysfunction models^15^. Moreover, LVEF of the cardiac dysfunction patients improved obviously after removing β_1_-AA by immunoadsorption (IA) treatment^16^. However, it is not elucidated about the underlying mechanism related to β_1_-AA-induced cardiac dysfunction. Our previous and other studies found that in β_1_-AA-positive murine, not only the cardiac function was decreased but accompanied by an increase in the peripheral CD4^+^/CD8^+^ T cell ratio; in addition, part of the myocardium was infiltrated by large number of T cells^17^. In vitro, β_1_-AA isolated from the sera of cardiac dysfunction patients promoted proliferation of CD4^+^ T cells through the β_1_-AR/cAMP pathway^14^. Furthermore, accompanied by cardiac function improvement of the β_1_-AA-positive cardiac dysfunction after IA treatment, the number of circulating nTreg cells increased significantly^18,19^. It was shown that nTreg cell proportion in rat peripheral blood was inhibited by β_1_-AR blocker propranolol^20^. However, whether β_1_-AA as a agonist-like substance of β_1_-AR can exert a direct effect on nTreg cells has not been reported.

Therefore, the present study was intended to assess the potential impact of β_1_-AA on nTreg cell activation and differentiation, and the underlying mechanism was explored in an attempt to etiologically find a potential therapeutic target for β_1_-AA-positive cardiac dysfunction patients.

## Results

### Activation of circulating nTreg cells in mice was promoted by β_1_-AA

**Fig. 1:**
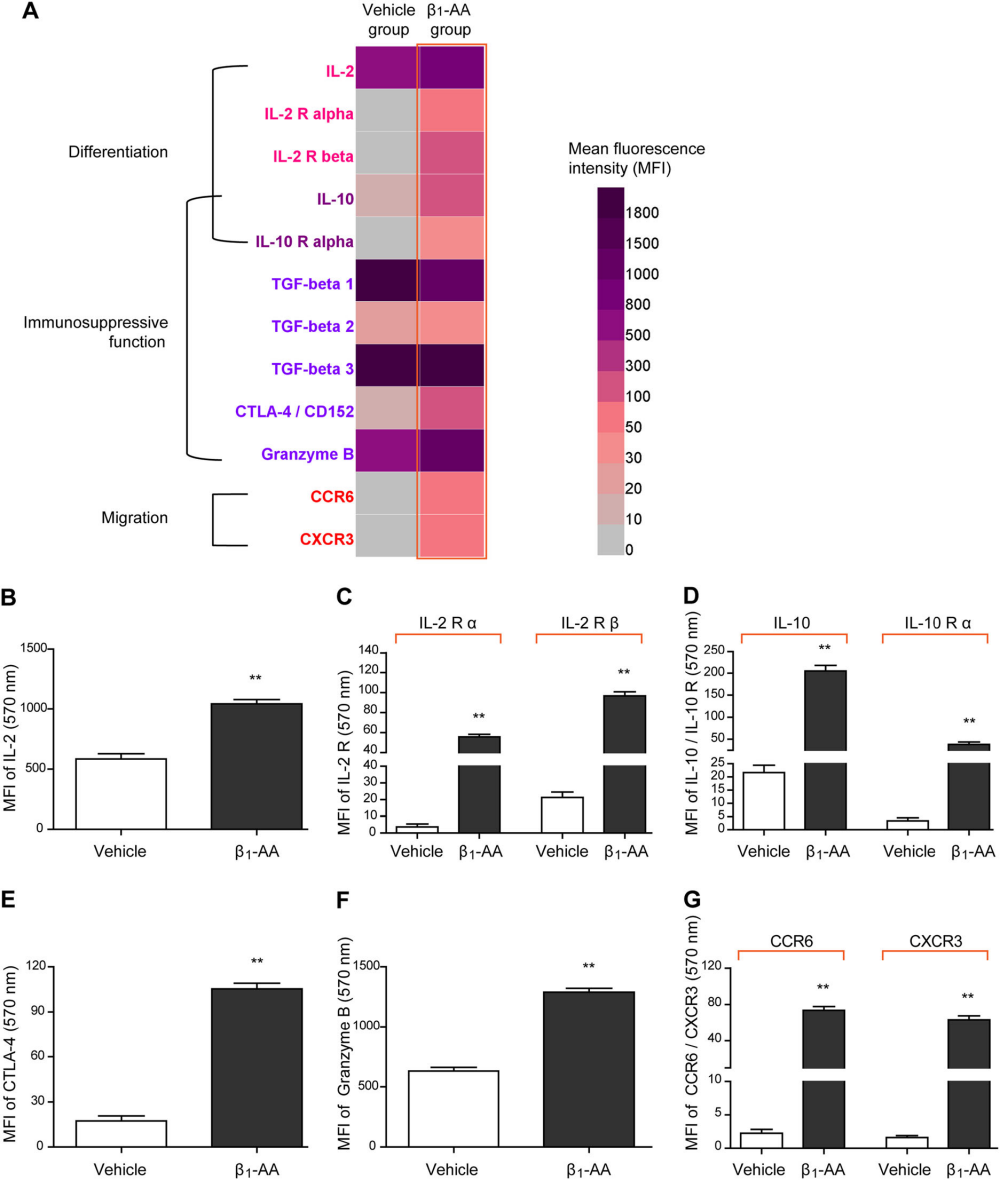
Expressions of nTreg cell-related proteins and cytokines in β_1_-AA positive mice. **a** Heat map of cluster analysis for expressions of nTreg cell-related proteins in β_1_-AA-positive mice at the eighth week after β_1_-AR mAb administration. Dark purple and light gray represented high and low levels of expression for the indicated proteins, respectively. All cytokines and cytokine receptors were detected by protein microarray chip analysis. **b–g** The quantitative levels of serum IL-2 (**b**), IL-2 receptor (**c**), IL-10/IL-10 receptor (**d**), CTLA-4 (**e**), granzyme B (**f**), and CXCR3 and CCR6 (**g**) in the sera of the model mice. Data are presented as means ± SD (*n* = 3 per group). **P *<* 0.01 vs. vehicle group

After 8 weeks β_1_-AR monoclonal antibody (β_1_-AR mAb) administration, optical density (OD) value of serum β_1_-AA was increased in mice, indicating that β_1_-AA-positive model was created successfully (Supplemental Fig. 1). Using the protein microarray chip technique, the expressions of nTreg cell-related proteins and cytokines were detected in β_1_-AA-positive mice at the eighth week after β_1_-AR mAb administration. The heat map of cluster analysis (Fig. 1a) showed that the expressions of interleukin-2 (IL-2)/IL-2 receptor (Fig. 1b, c), IL-10/IL-10 receptor (Fig. 1d), cytotoxic T-lymphocyte antigen 4 (CTLA-4) (Fig. 1e), granzyme B (Fig. 1f), chemokine receptor 3 (CXCR3) (Fig. 1g), and chemokine receptor CCR6 (Fig. 1g) in the sera of β_1_-AA-positive mice were enhanced significantly as compared with those in the vehicle group, of which IL-2 is known to be crucial for nTreg cell proliferation and differentiation^21,22^. IL-10^3^, granzyme B, and CTLA-4^23^ are known as important regulators in mediating the immunosuppressive activity of nTreg cells, while CCR6 and CXCR3 molecules are closely associated with nTreg cell recruitment^24,25^. Above all, β_1_-AA could promote nTreg cell activation in mice by up-regulating proteins related to nTreg cell differentiation, immunosuppressive function, and migration.

### Differentiation of nTreg cells from activated CD4^+^ T cells was facilitated by β_1_-AA

**Fig. 2:**
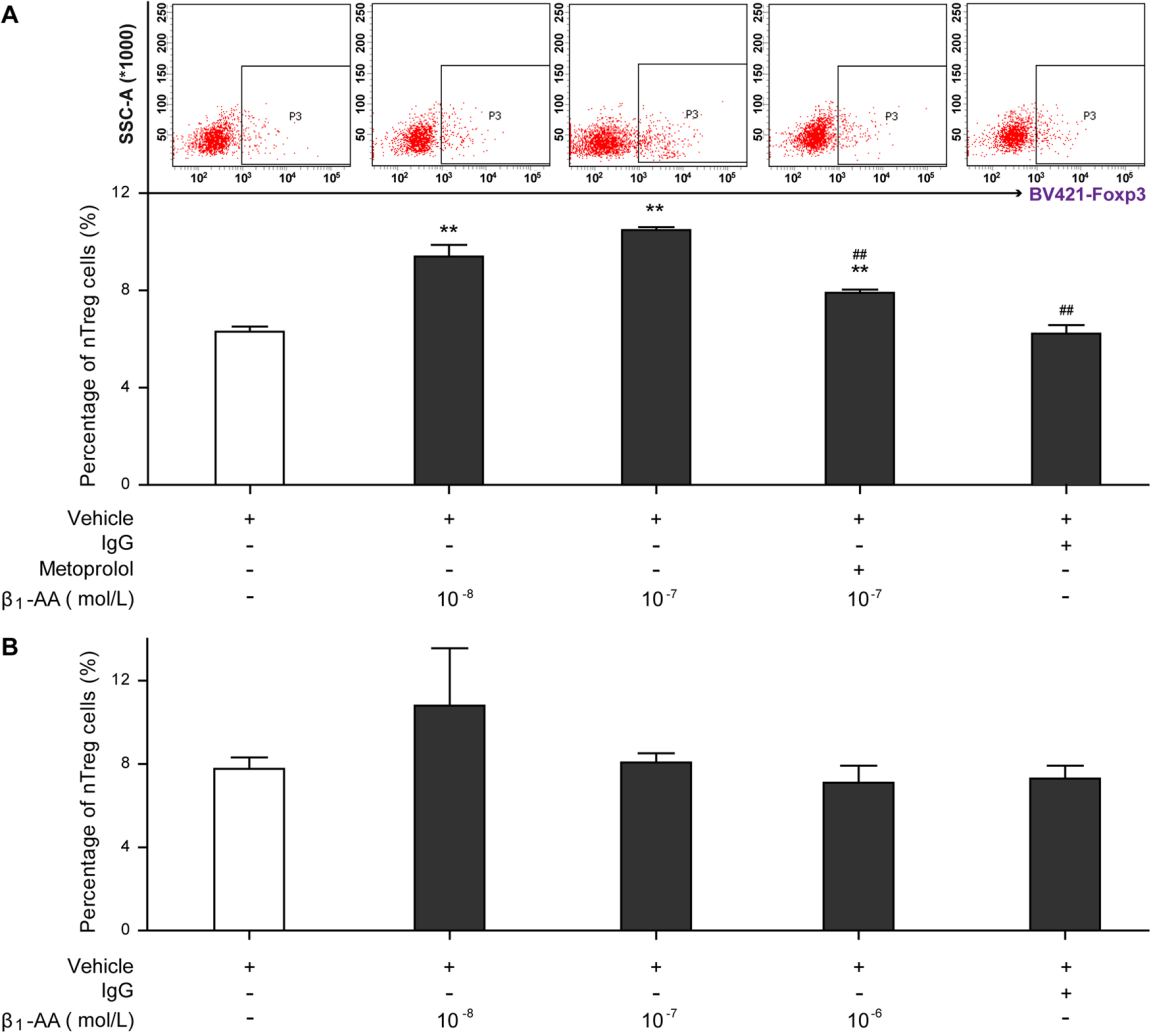
The proportion of nTreg cells among CD4^+^ T cells after β_1_-AA stimulation. **a** Percentage of nTreg cells in the preactivated CD4^+^ T cells at the 24th hour after β_1_-AA stimulation with or without metoprolol. **b** Percentage of nTreg cells in quiescent CD4^+^ T cells at the 24th hour after β_1_-AA stimulation. Data are presented as means ± SD (*n* = 5 per group). **P *<* 0.01 vs. vehicle group; ^*##*^*P* *<* 0.01 vs. 10^−8^ mol/L β_1_-AA group

CD4^+^ T cells sorted from the splenocytes of healthy mice were with or without preactivation by anti-CD3/CD28 mAbs (1 μg/mL) for 72 h, and then stimulated with different concentrations of β_1_-AA (10^−8^, 10^−7^, or 10^−6^ mol/L). Flow cytometry analysis showed that the proportion of nTreg cells among the preactivated CD4^+^ T cells was increased significantly 24 h after β_1_-AA administration (Fig. 2a). In addition, this effect can be reversed drastically by the β_1_-AR-specific blocker metoprolol (10^−7^ mol/L) (Fig. 2a). However, β_1_-AA was unable to promote the differentiation of nTreg cells from quiescent CD4^+^ T cells (Fig. 2b, *P* > 0.05).

### A metabolic shift toward fatty acid metabolism was associated with the increased nTreg cell differentiation induced by β_1_-AA

**Fig. 3:**
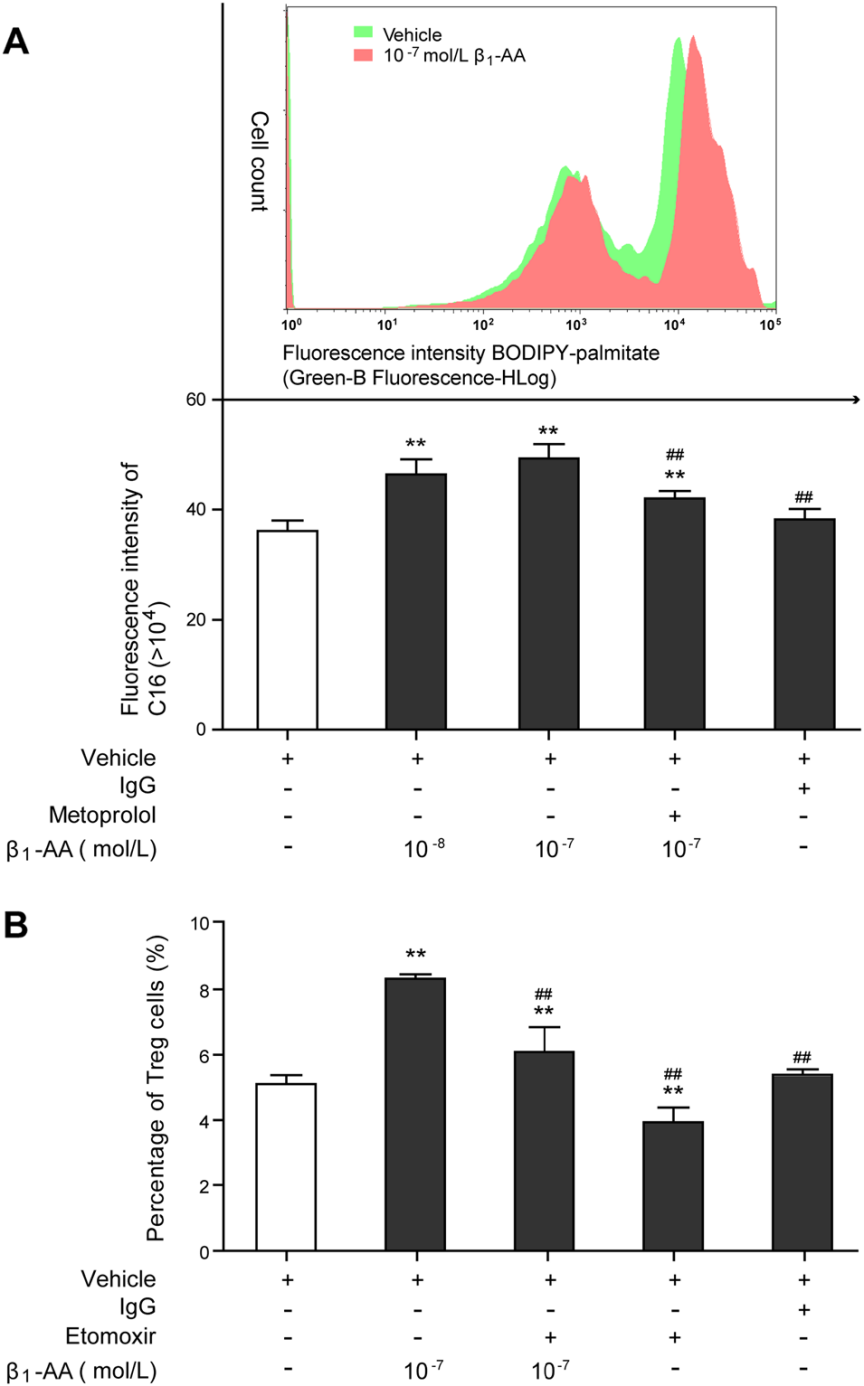
Influence of β_1_-AA on FAO in activated CD4^+^ T cells. **a** The absorption level of palmitate in activated CD4^+^ T cells after β_1_-AA stimulation with or without metoprolol. **b** Percentage of nTreg cells in activated CD4^+^ T cells after β_1_-AA stimulation with or without Etomoxir. Data are presented as means ± SD (*n* = 5 per group). **P < 0.01 vs. vehicle group; ^*##*^*P* *<* 0.01 vs. 10^−7^ mol/L β_1_-AA group

The mechanism underlying β_1_-AA-mediated nTreg differentiation was explored in the further experiments. Knowing that mitochondrial fatty acid oxidation (FAO) is a decisive factor for CD4^+^ T cell differentiation, which promotes CD4^+^ T cell differentiation towards Treg cells as opposed to an effector phenotype^26,27^. To assess whether FAO was affected by β_1_-AA in CD4^+^ T cells, the uptake of palmitate was measured in the course of stimulation. Flow cytometry demonstrated that the absorption of palmitate was increased in activated CD4^+^ T cells with anti-CD3/CD28 mAbs after β_1_-AA stimulation (Fig. 3a). Therefore, we postulated that the metabolic alteration in activated CD4^+^ T cells induced by β_1_-AA participated in enhanced nTreg cell differentiation. Etomoxir, a selective inhibitor of carnitine palmitoyltransferase I^28^, was used to confirm our hypothesis. Indeed, it was found that etomoxir was able to reverse the effect of β_1_-AA in nTreg cell differentiation (Fig. 3b). Thus, the enhanced nTreg cell differentiation elicited by β_1_-AA is accompanied by a metabolic shift toward FAO in activated CD4^+^ T cells and is reversible by FAO inhibitor.

### Activation of AMPK positively regulated nTreg cell differentiation induced by β_1_-AA

**Fig. 4:**
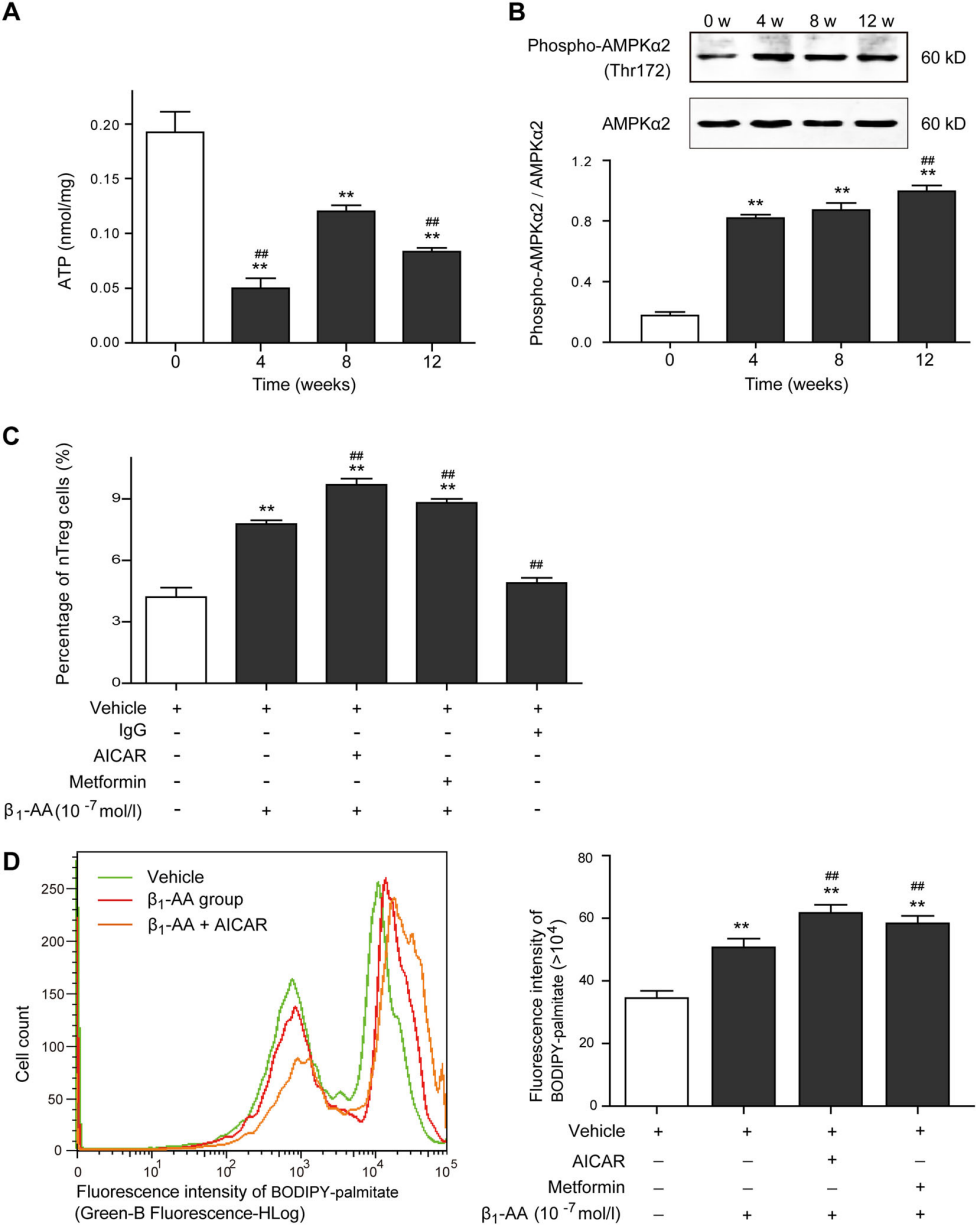
The role of AMPK in β_1_-AA-induced nTreg cell differentiation. ATP levels (**a**) and Thr(172)-AMPKα phosphorylation (**b**) were estimated in the primary CD4^+^ T cells isolated from the splenic tissue of β_1_-AA-positive mice at different time points during β_1_-AR mAb administration (*n* = 4 per group). **c** Percentage of nTreg cells in activated CD4^+^ T cells after β_1_-AA stimulation with or without AICAR (*n* = 5 per group). **d** The absorption level of palmitate in activated CD4^+^ T cells after β_1_-AA stimulation with or without AICAR/metformin (*n* = 5 per group). Data are presented as means ± SD. **a**, **b**: **P *<* 0.01 vs. 0 week since β_1_-AR mAb administration; ^*##*^*P* *<* 0.01 vs. the eighth week. **c**, -**d** **P < 0.01 vs. vehicle group; ^*##*^*P* *<* 0.01 vs. β_1_-AA group

AMP-activated protein kinase (AMPK) activation is known to enhance mitochondrial FAO in response to decreased ATP level^29^, which is a crucial pathway attributable to nTreg cell differentiation^27,30^. AMPK is mainly activated by increased AMP level and phosphorylation of a threonine residue (Thr-172)^31^. To investigate the role of AMPK in β_1_-AA-induced nTreg cell differentiation, AMPK phosphorylation and ATP levels were estimated in the primary CD4^+^ T cells isolated from the splenic tissue of β_1_-AA-positive mice. Significant decrease in ATP levels has been observed in the primary CD4^+^ T cells since the fourth week of β_1_-AR mAb administration until the 12th week (Fig. 4a), accompanied by constantly increased Thr(172)-AMPKα phosphorylation (Fig. 4b). Unlike the expression changes in the inhibitory phosphorylation site serine 491^32^, which increased at the fourth week, and then decreased at the eighth week (Supplemental Fig. 2), Thr(172)-AMPKα phosphorylation have been constantly increased since the fourth week of β_1_-AR mAb administration until the 12th week. What is more, the direct AMPK activator, 5-aminoimidazole-4-carboxamide riboside (AICAR) facilitated β_1_-AA-mediated nTreg cell differentiation in vivo (Fig. 4c) and promoted palmitate absorption in activated CD4^+^ T cells with anti-CD3/CD28 mAbs (Fig. 4d). Metformin, known as a indirect activator of AMPK by lowering the energy supply^30^, exhibited similar effects on β_1_-AA-mediated nTreg cell differentiation and palmitate absorption (Fig. 4c–d).

**Fig. 5:**
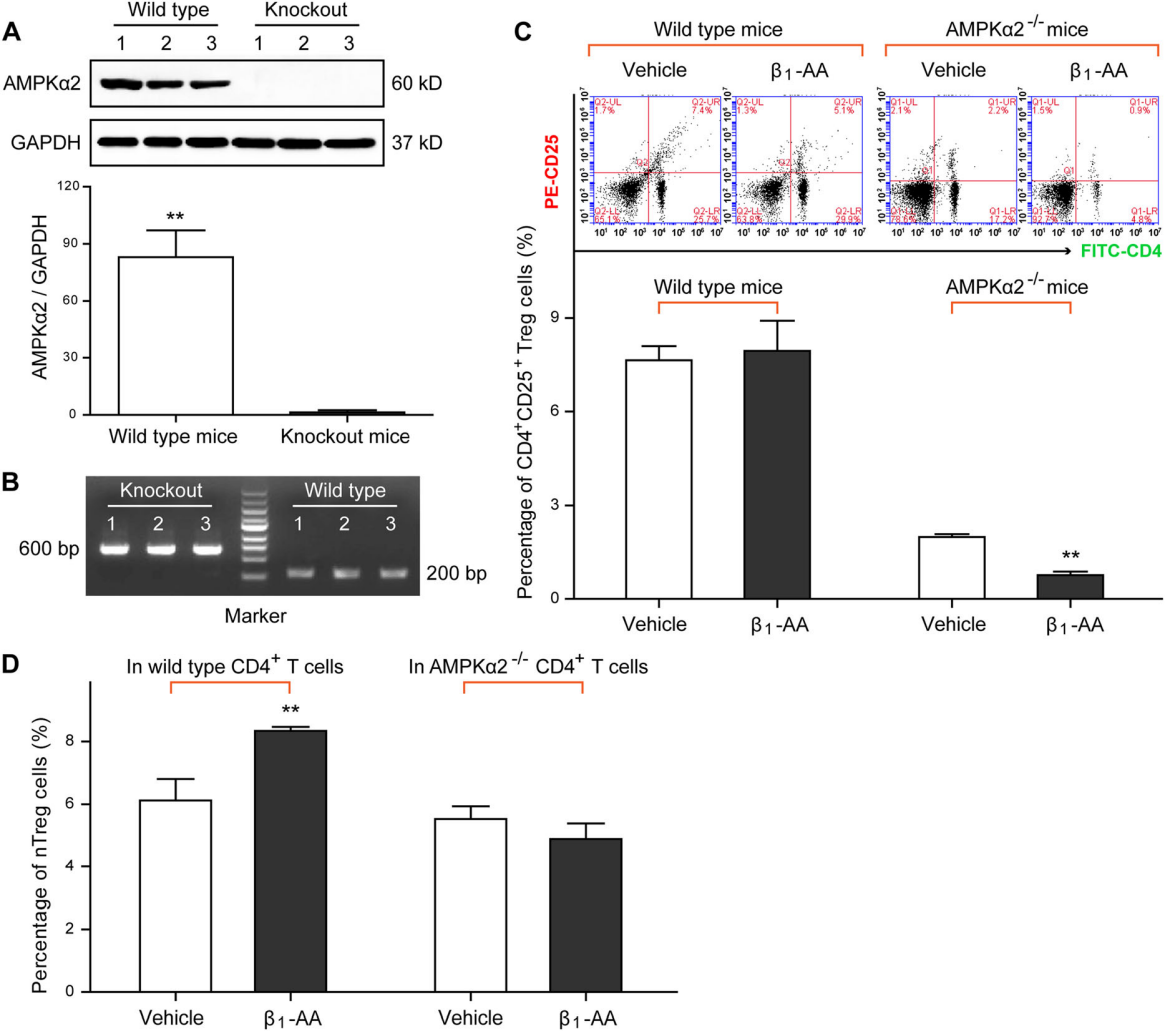
Influence of the knockout of the AMPKα2 gene in β_1_-AA-induced nTreg cell differentiation. **a** AMPK expression in AMPKα2^−/−^ mice at the protein level (*n* = 3 per group). **b** Identification of AMPKα2^−/−^ mice by gel electrophoresis on a 1.5% agarose gel. The result showed that two straps of 600 and 200 bp appeared, which represented the AMPKα2^−/−^ and wild-type C57BL/6 mice. **c** Frequency of the CD4^+^ CD25^+^ Treg cells in mice peripheral blood at the fourth week after β_1_-AR mAb administration (*n* = 6 per group). **d** Percentage of nTreg cells in activated AMPK^−/−^ CD4^+^ T cells or wild-type CD4^+^ T cells after β_1_-AA stimulation (10^−7^ mol/L) for 24 h (*n* = 5 per group). Data are presented as means ± SD. **a** **P < 0.01 vs. knockout mice; **c,** **d**: **P *<* 0.01 vs. vehicle group

To further explore the role of AMPK in β_1_-AA-induced nTreg cell differentiation, β_1_-AA was utilized on the CD4^+^ T cells isolated from AMPK-deficient (AMPKα2^−/−^) mice (Fig. 5a, b). Percentage of the circulating CD4^+^ CD25^+^ Treg cells in AMPKα2^−/−^ mice was lower than that in wild=type mice, and it was further reduced by 4-week β_1_-AA administration (Fig. 5c). In vitro, the result showed that nTreg cell proportion in the preactivated AMPK^−/−^ CD4^+^ T cells was lower than that in CD4^+^ T cells isolated from wild-type mice after β_1_-AA stimulation (10^−7^ mol/L) for 24 h (Fig. 5d). The evidence suggesting that knockout of the AMPKα2 gene decreased the effect of β_1_-AA in promoting nTreg cell differentiation. To sum up, these data demonstrate that AMKP activation plays a moderate positive role in nTreg cell differentiation mediated by β_1_-AA.

### Enhancement of fatty acid metabolism mediated by MMP reduction promoted β_1_-AA-induced nTreg cell differentiation

**Fig. 6:**
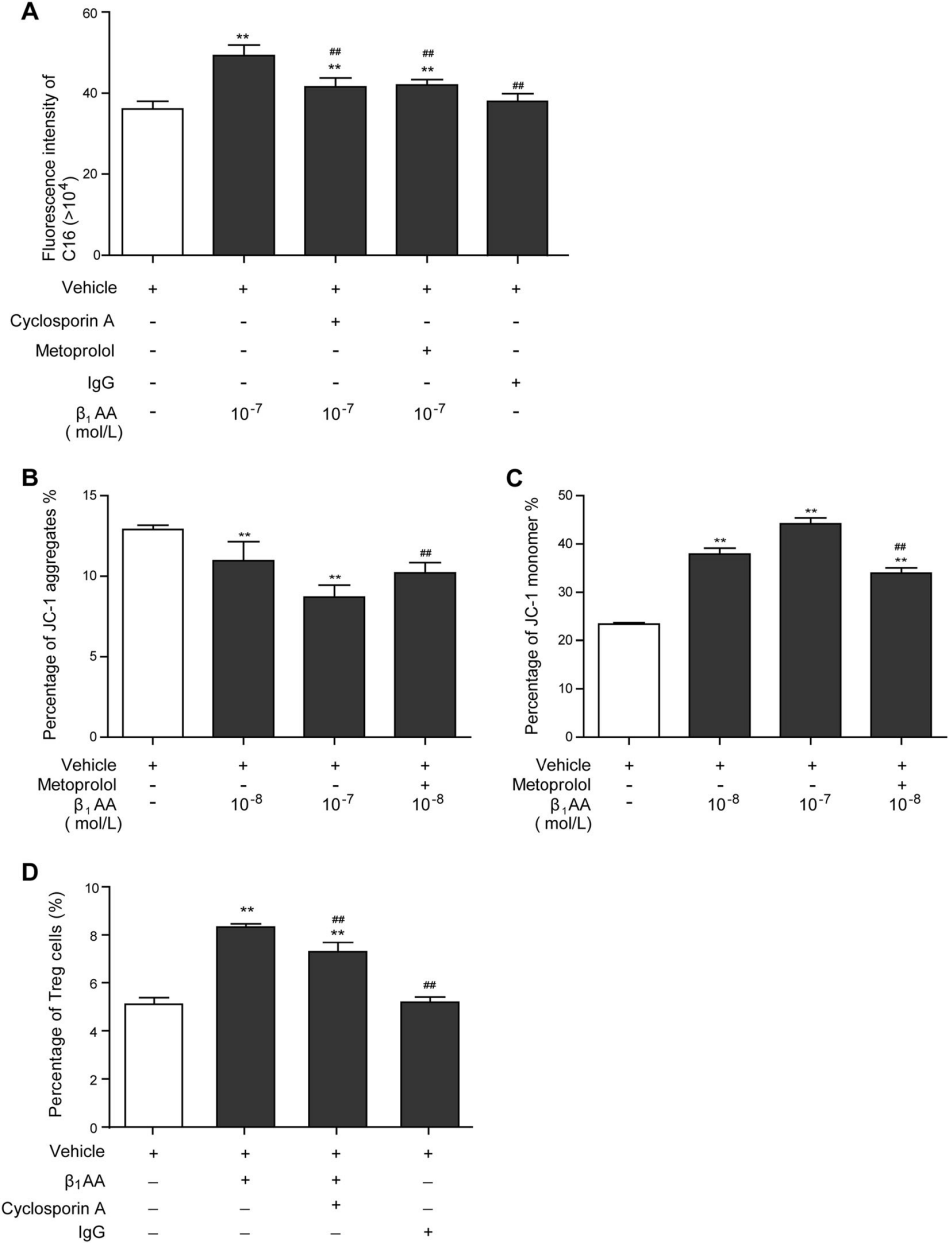
Effect of mitochondrial membrane potential (MMP) on β_1_-AA-induced nTreg cell differentiation. **a** The absorption level of palmitate in activated CD4^+^ T cells after β_1_-AA stimulation with or without cyclosporin A. **b**, **c** Percentages of JC-1 aggregate and JC-1 monomer in activated CD4^+^ T cells after β_1_-AA stimulation. **d** Percentage of nTreg cells in activated CD4^+^ T cells after β_1_-AA stimulation with or without cyclosporin A. Data are presented as means ± SD (*n* = 5 per group). **P < 0.01 vs. vehicle group; ^*##*^*P* < 0.01 vs. β_1_-AA group

The mitochondrial FAO is closely associated with mitochondrial membrane potential (MMP) level. When MMP is reduced, intracellular absorption and utilization of palmitate are enhanced, and FAO is up-regulated^33,34^. To determine whether β_1_-AA-induced nTreg cell differentiation resulted from MMP alteration in CD4^+^ T cells, cyclosporin A, an MMP stabilizer, was used in the course of β_1_-AA stimulation. It was found that the enhanced palmitate absorption elicited by β_1_-AA in activated CD4^+^ T cells with anti-CD3/CD28 mAbs was reversed drastically by cyclosporin A and metoprolol (Fig. 6a). As shown by JC-1 staining, a concurrent reduction in the MMP of preactivated CD4^+^ T cells was found in the β_1_-AA group (Fig. 6b, c). Subsequently, cyclosporin A inhibited the elevation of nTreg cell differentiation mediated by β_1_-AA (Fig. 6d). Therefore, enhanced fatty acid metabolism mediated by MMP reduction in preactivated CD4^+^ T cells is one of the mechanisms underlying nTreg cell differentiation induced by β_1_-AA.

## Discussion

β_1_-AA was first identified in the sera of patients with dilated cardiomyopathy by Wallukat et al. in 1987^35^. Subsequently, ample evidence has confirmed the pathogenic effect of β_1_-AA in cardiac dysfunction^9,11,15^. However, both our study and others found that β_1_-AA was positive in about 10% of healthy individuals of different age groups^36–38^, suggesting that β_1_-AA may participate in maintaining physiological homeostasis, although the correlative mechanism is unclear. nTreg cells play a very important role in maintaining the balance of the immune system by inhibiting effector T cells^2,3^, and their differentiation and function are regulated by the sympathetic nervous system^7,8^. Nevertheless, whether β_1_-AA as a agonist-like substance of β_1_-AR, could exert a direct effect on nTreg cells has not been reported. Therefore, the present study sought to assess the potential impact of β_1_-AA on nTreg cell differentiation and explore the underlying mechanism. It was found that β_1_-AA promoted nTreg cell differentiation by up-regulating fatty acid metabolism in activated CD4^+^ T cells via the AMPK pathway and MMP reduction.

To study the effect of β_1_-AA on Treg cells, a passive immunization mouse model was established successfully with the highly active and purified β_1_-AR mAb (Supplemental Fig. 3). Naïve CD4^+^ T cells differentiate into different subsets (Th1, Th2, Th17, or Treg cells) to establish immune tolerance and defense against pathogens. To quantify CD4^+^ T cell-related cytokine levels, the levels of Th1 cytokine (IFN-γ), Th2 cytokine (IL-4), Th17 cytokine (IL-17), and Treg cytokine (IL-10) in the sera of β_1_-AA-positive mice was assessed by a bead-based multianalyte flow assay kit. Quantitative analysis demonstrated that levels of Treg cytokine (IL-10) (Supplemental Fig. 4A), Th17 cytokine (IL-17) (Supplemental Fig. 4B), Th1 cytokine (IFN-γ) (Supplemental Fig. 4C), and Th2 cytokine (IL-4) (Supplemental Fig. 4D) increased in mice 8 weeks after β_1_-AA administration, indicating that β_1_-AA promoted a systemic activation of CD4^+^ T cell in vivo. What is more, the expression of multiple proteins related to nTreg cell differentiation, immunosuppressive function, and migration increased in mice peripheral blood, suggesting that β_1_-AA was able to promote nTreg cell activation. However, ultrasound analysis showed that β_1_-AA-induced cardiac dysfunction in mice, as illustrated by decreases in LVEF (Supplemental Fig. 5A), fractional shortening (Supplemental Fig. 5B), and cardiac output (Supplemental Fig. 5C), accompanied with a decreased proportion of circulating CD4^+^ CD25^+^ Treg cells (Supplemental Fig. 6). These results are consistent with the finding of many other studies that Treg cell frequency in cardiac dysfunction patients was decreased significantly^39–41^, and the number of Treg cells was positively correlated with LVEF, and negatively correlated with the NT-proBNP level^42^. Nevertheless, increased Treg cell infiltration was observed in the myocardium of mice with cardiac dysfunction^43^. For this reason, we explored whether β_1_-AA had a direct effect on nTreg cell differentiation in our subsequent experiment in vitro.

It was found that β_1_-AA induced a metabolic shift towards FAO in activated CD4^+^ T cells, thus promoting nTreg cell differentiation. In addition, the effect of β_1_-AA in promoting nTreg cell differentiation could be reversed drastically by the β_1_-AR-specific blocker metoprolol. Other studies also demonstrated that atecholamines such as epinephrine and norepinephrine increased the proportion of Treg cells, and the β_1_-AR blocker propranolol attenuated such elevation of Treg cells^44^. Regulation of metabolism decides the fate of CD4^+^ T cell differentiation, and FAO is crucial in inducing Treg cell differentiation vs. Teff cell lineages^26,27,45^. In other words, Treg cell differentiation depends on FAO, and we found that the FAO inhibitor etomoxir reversed β_1_-AA-mediated nTreg cell differentiation. MMP which effects fatty acid uptake^33,34^ and AMPK activation^29,30,46^ are two pivotal regulator for FAO. Furthermore, the underlying mechanism involved in β_1_-AA-mediated nTreg cell differentiation was explored.

Indeed, β_1_-AA reduced MMP of activated CD4^+^ T cells, and the MMP stabilizer cyclosporin A drastically reversed β_1_-AA-induced fatty acid absorption enhancement and nTreg cell differentiation. Our previous study^29,30,47^ demonstrated that β_1_-AA-induced cardiomyocyte apoptosis by reducing MMP. Similarly, the present study demonstrated that the level of CD4^+^ T cell apoptosis was increased significantly after β_1_-AA stimulation shown by Annexin V-FITC and propidium iodide double staining (Supplemental Figure 7). These findings suggest that β_1_-AA promoted nTreg cell differentiation through up-regulating FAO and reducing MMP, and this effect is closely associated with β_1_-AA-induced CD4^+^ T cell apoptosis. In addition, MMP reflects the integrity of mitochondrial function and is a key indicator of mitochondrial function^48^. The mitochondrial function alteration may participate in nTreg cell differentiation elicited by β_1_-AA.

AMPK is a key regulatory molecule in response to energy deprivation of the organism^29^. It provides energy quickly by promoting FAO and inhibiting the activity of acetyl coenzyme A carboxylase^49,50^. AMPK-dependent metabolic regulation plays an important role in Treg cell differentiation^30,46^. We found that the ATP level in the primary CD4^+^ T cells isolated from the splenic tissue of β_1_-AA positive mice was significantly lower than that in the vehicle group, which was accompanied by enhanced Thr(172)-AMPKα phosphorylation. It was reported that AICAR, a pharmacological analog of AMPK^30,46^, promoted Treg cell differentiation without affecting effector T cells^51^. Besides, norepinephrine induced AMPK activation via the cAMP/β-AApathway^52^. The present study showed that both the direct AMPK activator AICAR and the indirect AMPK activator metformin facilitated β_1_-AA-mediated FAO enhancement and nTreg cell differentiation in vitro. Moreover, AICAR promoted IL-2 level in the supernatant of activated CD4^+^ T cells after β_1_-AA stimulation, which is crucial for nTreg cell differentiation (Supplemental Figure 8). To further confirm the role of AMPK in β_1_-AA-mediated nTreg cell differentiation, β_1_-AA was acted on the CD4^+^ T cells isolated from the AMPKα2^−/−^ mice. It was found that knockout of the AMPKα2 gene reduced the effect of β_1_-AA in promoting nTreg cell differentiation markedly, confirming that AMPK-induced FAO is a key mechanism underlying β_1_-AA-mediated nTreg cell differentiation.

According to the published paper^52,53^, the β_2_-AR/cAMP/PKA pathway played a positive moderate role in the immunosuppressive activity of Treg cells. Indeed, cAMP levels increased in the supernatants of nTreg cells after 30-min β_1_-AA stimulation, which is also the downstream signal molecule of β_1_-AR (Supplemental Figure 9A). However, immunofluorescence staining showed that the fluorescein-labeled β_1_-AA was incapable of binding to β_2_-ARs on Treg cells compared to the anti-β_2_-AR mAb (Supplemental Figure 9B). Moreover, by contrast to the activation effect of β_2_-AR pathway, proliferation assay of the CD4^+^ CD25^−^ T effector cells revealed that compromised suppressive activity of nTreg cells were resulted from 48h β_1_-AA administration (Supplemental Figure 10). The inhibitory effect of nTreg cells on Teff cells is mainly mediated by IL-10 production^54^. In view of the fact that nTreg cell dysfunction may induced by β_1_-AA, IL-10 level was measured in the supernatant of nTreg cells after β_1_-AA stimulation. It was found that IL-10 secretion from nTreg cells was suppressed by β_1_-AA at concentrations of 10^−6^ and 10^−7^ mol/L compared with vehicle groups (Supplemental Figure 11). However, 10^−8^ mol/L β_1_-AA had a remarkable opposite effect, and 10^−9^ mol/L β_1_-AA did not appear to be a factor (Supplemental Figure 11). The evidences above indicated that β_1_-AA had bidirectional impact on the immunosuppressive function of nTreg cells. Yet, the decline in cardiac function induced by β_1_-AA (Supplemental Fig. 5) seems to outweigh the potentially beneficial effects on nTreg cell restoration. Nevertheless, like many other physiological processes, the influence of β_1_-AA on organism is a double-edged sword with therapeutic potential that is associated with the concentration of β_1_-AA.

### Limitation and clinical perspective

Till now, the influence of β_1_-AR gene knockout on Treg maturation and function has not been reported. In order to investigate the role of β_1_-AR in β_1_-AA-induced nTreg differentiation, our lab had already acquired three pairs of homozygous β_1_-AR gene knockout mice (C57BL/6J background) from Nanjing BioMedical Research Institute of Nanjing University recently. However, yet the number of available transgenic mice is not sufficient to build our model.

Cardiac dysfunction associated with myocardial injury triggers β_1_-AA generation in different cardiac dysfunction models, and the positive rate of β_1_-AA is nearly 80%^14^. However, there is no specific and effective therapeutic strategy for β_1_-AA-positive patients. β_1_-AR blockers cannot entirely reverse the injurious effect of β_1_-AA on cardiomyocytes^55,56^. The present study showed that the impacts of β_1_-AA on nTreg cell differentiation cannot be fully counteracted by β_1_-AR-specific blocker metoprolol (Fig. 2a, Fig. 3a, and Fig. 6a–c), indicating that there are other mechanisms involved except for a receptor pathway. It is therefore an urgent task to find a more effective therapeutic target specific for β_1_-AA-positive cardiac dysfunction patients. The present study demonstrated that AMPK-mediated metabolic regulation targeting for nTreg cell restoration might be a promising therapeutic target for β_1_-AA-positive cardiac dysfunction patients.

## Materials and methods

### Synthesis and identification of β_1_-AR mAb

The sequence (amino-acid residues 197–222) of the second extracellular loop of the β_1_-AR: H-W-W-R-A-E-S-D-E-A-R-R-C-Y-N-D-P-K-C-C-D-F-VT-N-R-C was synthesized by solid-phase method using an automated peptide synthesizer. Subsequently, 0.5 mg synthetic polypeptide was coupled with the carrier protein keyhole limpet hemocyanin and bovine serum albumin (BSA) to acquire immunogenicity. The coupled polypeptide was applied to BALB/c mice to create active immunization and induce the production of β_1_-AR-ECII-specific antibodies. Finally, these specific antibodies were fused with the hybridoma cell line to synthesize mAbs specific to β_1_-AR-ECII. The synthesis of β_1_-AR-ECII peptide was conducted by Qiang Yao Bio Scientific Commercial Development Co., Ltd (Shanghai, China), and the hybridoma cells secreting β_1_-AR mAb were constructed by AbMax Biotechnology Co., Ltd (Beijing, China).

To induce the generation of ascites containing β_1_-AR mAb, log-phase hybridoma cells were injected intraperitoneally to female BALB/c mice aged 10 weeks at a dose of 10^6^ cells per mL, 0.5 mL per mouse biweekly. The ascites was collected and then purified by using Protein G Affinity Chromatography Column (GE Healthcare Life Sciences, USA). The specificity and activity of the purified β_1_-AR mAbs were determined by enzyme-linked immunosorbent assay (ELISA) and the neonatal mouse cardiomyocyte beating experiment, respectively.

### ELISA

The specificity of the purified β_1_-AR mAb and the OD value of β_1_-AA in mice serum were detected by ELISA. Briefly, the β_1_-AR-ECII peptide was dissolved in 100 mM 10 µg/mL Na_2_CO_3_ solution (pH = 11.0) at 4 °C overnight. The embedded 96-well plate was incubated with 1% BSA at 37 °C for 1 h, and then cultured with the primary antibody. Biotin-labeled anti-mouse immunoglobulin G (IgG) was diluted with the sealing solution at a ratio of 1:3000 and cultured at 37 °C for 1 h. Horseradish enzyme-labeled streptavidin was diluted at a ratio of 1:2000 and cultured at 37 °C for 1 h. The substrate ABTS (2,2′-azino-di-(ethyl-benzthiazoline) sulfonic acid) was dissolved in the substrate buffer with a final concentration of 1.1 mmol/L and cultured at 37 °C for 30 min. The optical density (OD) value of each well was measured at 405 nm. The titer of β_1_-AR mAb was determined by the positive/negative (P/N) ratio using the following equation: P/N = (sample OD–blank control OD)/(positive control OD–blank control OD). The positivity or negativity of β_1_-AA was determined by P/N ≥ 2.1 or P/N ≤ 1.5, respectively.

### Establishment of the β_1_-AA-positive mouse model

Male C57BL/6 mice aged 8–10 weeks (weighing 19–28 g) were purchased from the Vital River Laboratory Animal Technology Co., Ltd (Beijing, China). Homozygous AMPKα2-deficient (AMKPα2^−/−^) mice in the C57BL/6 background (weighing 18–26 g) were kindly provided by Dr. Benoit Viollet (Institute National de la Santé et de la Recherche Médicale U567, Paris). The genotype of the AMKPα2^−/−^ mice was evaluated by PCR using tail DNA. All the experimental mice were housed under the 26 ± 1.5 °C, 40–60% humidity and specific pathogen-free conditions (fewer than five animals in a cage). All animal experiments were performed according to the regulation for animal management issued by the Ministry of Health of the People’s Republic of China (Document No. 55, 2001), and approved by the ethics committee of the Capital Medical University (Beijing, China; Ethical number: AEEI-2016-013).

Twenty-four C57BL/6 mice which were β_1_-AA negative confirmed by ELISA were equally randomized to three groups: vehicle group, β_1_-AR mAb group, and negative IgG group. Mice in β_1_-AR mAb group received intraperitoneal injection of β_1_-AR mAb at a dose of 5 μg/g biweekly. Mice in the vehicle group received the same dose of normal saline, and the mice in the negative IgG group received the same dose of negative IgG.

### Protein microarray chip analysis

The expression of Treg cell-related proteins and cytokines in the β_1_-AA-positive mice was detected using a biotin-labeled mouse protein chip reagent kit. Briefly, each chip well was added with 100 µL sealing solution, cultured on the rocking bed for 30 min at room temperature. After sucking out the sealing solution, each well was added with 100 µL serum, cultured by oscillation at 4 °C overnight, and centrifuged at 13,000 rpm for 8 min. Each well was added with 70 µL biotin-labeled antibody and cultured at room temperature for 1 h after two washes of the plate. Then, 70 µL fluorant Cy3-streptomycin avidin was added to each well and cultured by oscillation at room temperature for 2 h. Finally, the fluorescent signal was detected by the Cy3 or green channel (532 nm).

### Flow cytometric sorting for CD4^+^ T cells

Specific fluorescent antibody-labeled CD4^+^ T cells were separated from mouse spleen mononuclear cells by flow cytometry. C57BL/6 mice aged 10 weeks were euthanized by cervical dislocation to isolate splenocytes. Then, a single-cell suspension was prepared by using mechanical trituration method where the tissue was ground through a 300-mesh sieve. Subsequently, mouse spleen mononuclear cells were re-suspended using 50% and 70% Percoll separating media (GE Healthcare Life Sciences, USA). Density-gradient centrifugation was undertaken at 2500 rpm for 25 min following red blood cell lysis. The steps mentioned above were performed at a fast pace, at 4 °C or on ice. For cell surface staining, the antibodies (FITC-anti-CD4) were incubated with the single-cell suspension for at least 30 min at 4 °C. FITC-anti-CD4 antibody was purchased from BD Bioscience (USA). Cells were sorted with the flow cytometer FACS Aria II (Becton, Dickinson and Company).

### Flow cytometric analysis for the proportion of nTreg cells

CD4^+^ T cells sorted from the splenocytes of healthy mice were with or without preactivation by anti-CD3/CD28 mAbs (1 μg/mL) (eBioscience, USA) for 72 h. Then, the cells were stimulated with different concentrations of β_1_-AA (10^−8^, 10^−7^, or 10^−6^ mol/L), with or without metoprolol (the β_1_-AR-specific blocker, 10^−8^ mol/L), for 24 h. Subsequently, CD4^+^ T cells were surface stained with anti-CD4/CD25 mAbs (FITC/phycoerythrin (PE)) for 30 min at 4 °C. After staining, cells were fixed and permeabilized using an intracellular fixation and permeabilization buffer set (eBioscience, USA), followed by intracellular staining with anti-FoxP3 mAb (BV421) (BD Bioscience, USA). The stained cells were centrifuged at 1500 rpm for 5 min, and re-suspended in 250 μL flow cytometric buffer solution (1% FBS in phosphate-buffered saline (PBS)). Flow cytometry was performed on the FACS Aria II flow cytometer (Becton, Dickinson and Company).

### Effect of β_1_-AA on CD4^+^ T cell absorption of palmitic acid and subsequent FAO

BODIP is a fat-soluble fluorescent probe. Coupling of BODIP and palmitate can be used to observe the cellular FAO level (Life Technologies, USA). A 5 mmol stock solution was prepared by dissolving the BODIPY–palmitate into dimethyl sulfoxide, and then diluted in PBS buffer to a working concentration of 0.5 µmol. All intervention factors and BODIPY–palmitate were added to the activated CD4^+^ T cells and cultured at 37 °C for 48 h. Finally, the fluorescent intensity of each tube was detected with the FACS Aria II flow cytometer (Becton, Dickinson and Company).

### Measurement of the ATP content

The ATP content in CD4^+^ T cells of β_1_-AA-positive mice was determined using a kit purchased from Beyotime Institute of Biotechnology (China) according to the manufacturer’s protocol.

### Western blotting

The expression of AMPK in CD4^+^ T cells was determined by Western blot analysis. CD4^+^ T cells were separated from the splenic tissues of the β_1_-AA-positive mice and immediately lysed. The supernatant protein was extracted by centrifugation. The supernatant protein was analyzed by sodium dodecyl sulfate-polyacrylamide gel electrophoresis at a 50 μg sample volume. After electrophoresis, the PVDF membranes were transferred and blocked with 5% non-fat milk powder in TBST buffer for 1 h, and then incubated with anti-AMPKα2 mAb (1:1000; Abcam, Cambridge, UK), anti-phospho-AMPKα2 (Thr-172) mAb (1:1000; Abcam, Cambridge, UK) or anti-phospho-AMPKα2 (S491) mAb (1:1000; Abcam, Cambridge, UK) or anti-GAPDH mAb (Cell Signaling Tech., Danvers, MA, USA) at 4 °C overnight. The membranes were incubated with the corresponding secondary antibodies. Finally, the grayscale values of the straps were analyzed by Image J software after development.

### Genotype identification of IL-10^−/−^ mice

The genotype of AMPKα2^−/−^ mice was identified by PCR of tail genomic DNA, using the following specific primers: 5′-GCT TAG CAC GTT ACC CTG GAT GG-3′ (forward, common), 5′-GCA TTG AAC CAC AGT CCT TCC TC-3′ (reverse 1, mutation), and 5′-GTT ATC AGC CCA ACT AAT TAC AC-3′ (reverse 2, wild type). Each 25-μl PCR mixture contained 20 pmol of each primer. The reaction conditions of PCR were as follows: 95 °C, 5 min; 94 °C, 30 s; 64 °C, 35 s, then 35 cycles of 72 °C, 45 s, followed by a final 3 min at 72 °C.

### JC-1 staining

When the cellular MMP remained stable, JC-1 aggregated in the mitochondrial matrix, producing red fluorescence; when MMP was reduced, JC-1 was present as a monomer in the cellular matrix, producing green fluorescence. Therefore, changes in MMP can be detected by observing the percentage of the red and green fluorescence. Briefly, 48 h after stimulation of activated CD4^+^ T cells (10^6^ cells per well) with β_1_-AA, cells were re-suspended in 0.5 mL RPMI medium 1640 (Hyclone, USA). After the addition of 0.5 mL JC-1 dye, cells were cultured in a 37 °C incubator for 20 min. Then, cells were re-suspended by the addition of 300 μL JC-1 staining buffer after being washed with JC-1 staining buffer in a centrifuge at 600 × *g* and 4 °C for 3 min twice. Finally, the red and green fluorescent intensity of each tube were detected and analyzed using the FACS Aria II flow cytometer.

### Statistical analysis

Data are presented as mean ± SD. Statistical analysis was performed with the SPSS Statistics software (version 16.0, SPSS Inc., Chicago, IL, USA). The differences between groups were analyzed using independent sample *t* tests, one-way or two-way analysis of variance (between different mice strains). Histograms were produced by GraphPad Prism 6 (GraphPad Software Inc., USA). A *P* value <0.05 was considered statistically significant. Differences in the heat map of cluster analysis were statistically significant when the fold change between two groups was >1.5.

## Electronic supplementary material

Supplemental methods and supplemental figures
