# Supplementary material for: Autoantibody against β1-adrenoceptor promotes the differentiation of natural regulatory T cells from activated CD4+ T cells by up-regulating AMPK-mediated fatty acid oxidation
Source: Cell Death Dis. 2019 Feb 15;10(3):158. doi: 10.1038/s41419-018-1209-2 (PMC6377640; doi:10.1038/s41419-018-1209-2)
Supplement: Supplementary file 1 — Supplemental methods and supplemental figures [file 41419_2018_1209_MOESM1_ESM.docx]

**SUPPLEMENTAL MATERIAL**

**Supplemental Methods**

**Analysis of cytokines level**

Concentrations of CD4^+^ T-cell related cytokines (IFN-γ, IL-4, IL-17 or IL-10) in mice serum were measured by a bead-based multi­analyte flow assay kit (BioLegend, San Diego, CA, USA) [under the](http://xueshu.baidu.com/s?wd=paperuri:(785dbac297a9e0cd7107f4829c214cfc)&filter=sc_long_sign&sc_ks_para=q=Comparison of test performance under the manufacturer's dilution protocol versus the 50% w/v protocol.&sc_us=3171780640275613076&tn=SE_baiduxueshu_c1gjeupa&ie=utf-8" \t "http://xueshu.baidu.com/_blank) manufacturer's recommended protocol. Four bead populations with distinct fluorescence intensities have been coated with capture antibodies specific for IFN-γ, IL-4, IL-17, or IL-10. Therefore, the four individual bead populations were mixed together during the assay preparation. Then, the cytokine capture beads were combined with the PE-conjugated detection antibodies and incubated with the recombinant standards or samples to form sandwich complexes. Following acquisition of the mean fluorescence intensity (MFI) by the BD LSRFortessa Flow Cytometry, the data of each sample were generated in a graphical format using the LEGENDplex v7.0 analysis software.

**T-cell proliferation assay**

A 5 mmol CellTrace™ CFSE (Life Technologies, New York, USA) stock solution was prepared by dissolving the CFSE into Dimethyl Sulfoxide (DMSO). The stock solution was diluted in PBS buffer to a working concentration of 0.5 µmol. Responder cells (CD4^+^ CD25^-^ T cells) were labeled with CFSE and incubated at 37 °C for 15 min in the dark. Subsequently, the staining process was stopped by adding RPMI 1640 medium (with 10 % FBS). After this, the responder cells were divided into the following groups: cultured with RPMI 1640 medium alone (with 10% FBS), co-cultured with the unlabeled nTreg cells or with 10^-7^ mol/L β_1_-AA administrated nTreg cells (at a ratio of 1:1). All cell groups were cultured in the presence of anti-CD3 mAb (3 μg/mL) for 72 h. Finally, the BD Accuri C6 Flow Cytometer and the FlowJo Software were used to quantify the proliferation ratios of responder cells in each group, respectively.

**Supplemental Figures**

**
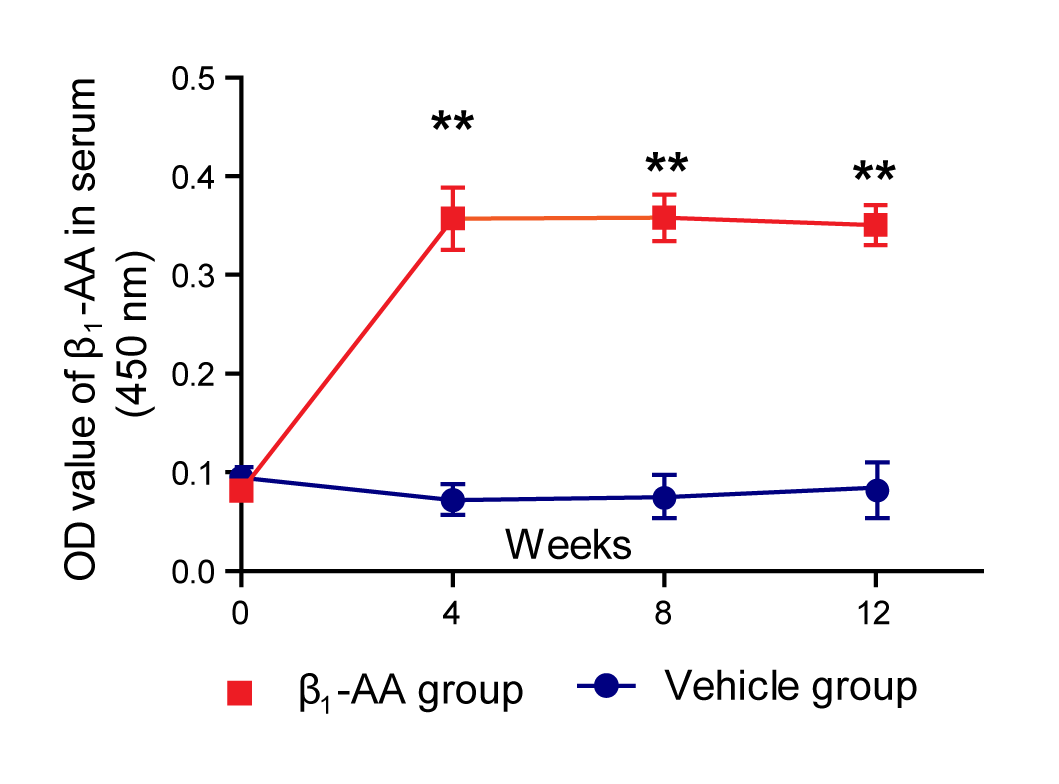
**

**Supplemental Figure 1:** Levels of β_1_-AA in mice sera during the process of β_1_-AR mAb passive immunization at different time points determined by SA-ELISA. OD value, Optical density value. Data are presented as means + SD (n = 8 per group). ***P* < 0.01 *vs.* vehicle group.

**
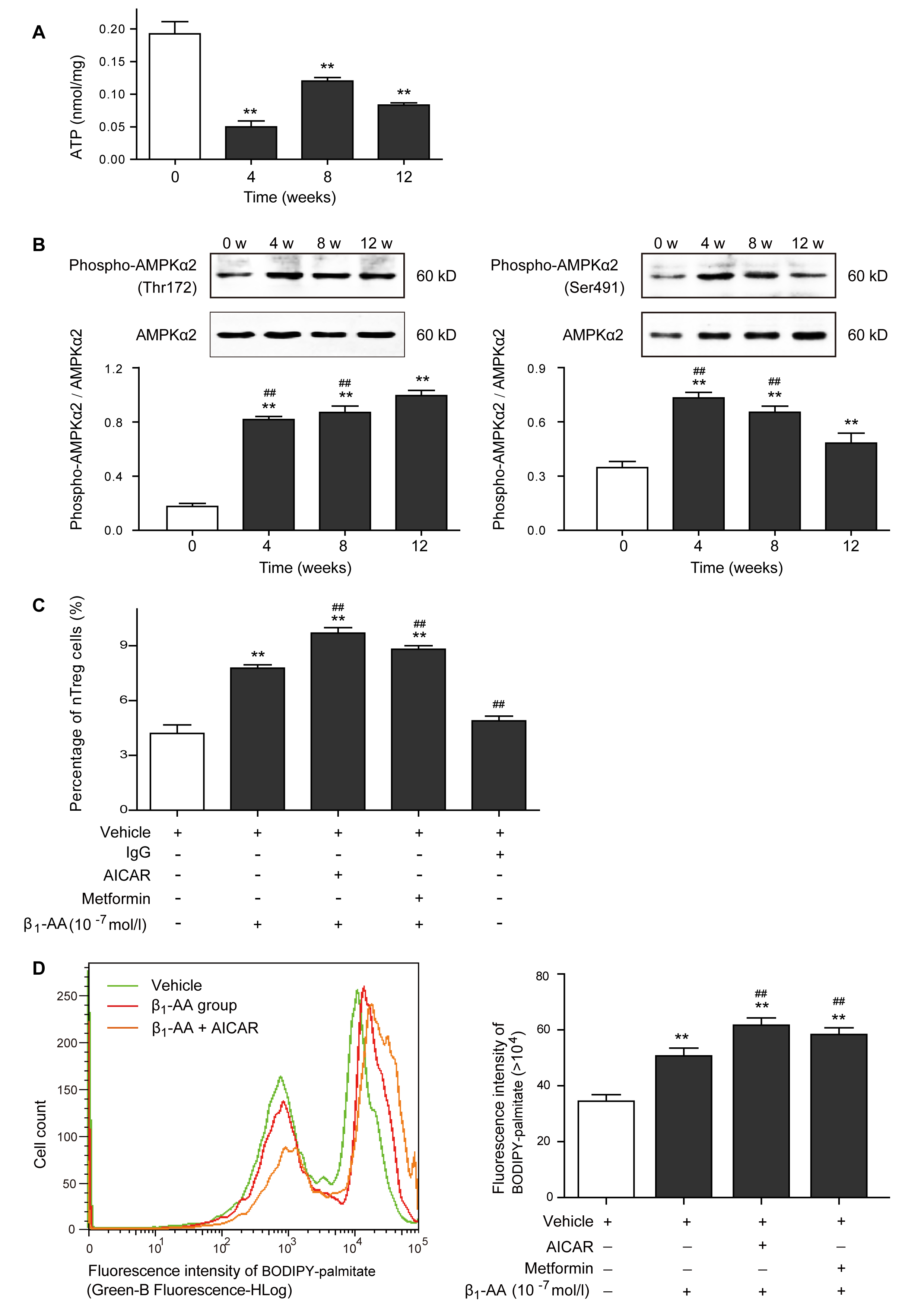
**

**Supplemental Figure 2:** Phosphorylation level of the Ser(491)-AMPKα was estimated in the primary CD4^+^ T cells isolated from the splenic tissue of β_1_-AA positive mice at different time points during β_1_-AR mAb administration (n = 4 per group). Data are presented as means + SD. A-B: ***P* < 0.01 *vs.* 0 week since β_1_-AR mAb administration; *^##^P* < 0.01 *vs.* The 12^th^ week of β_1_-AR mAb administration.

**
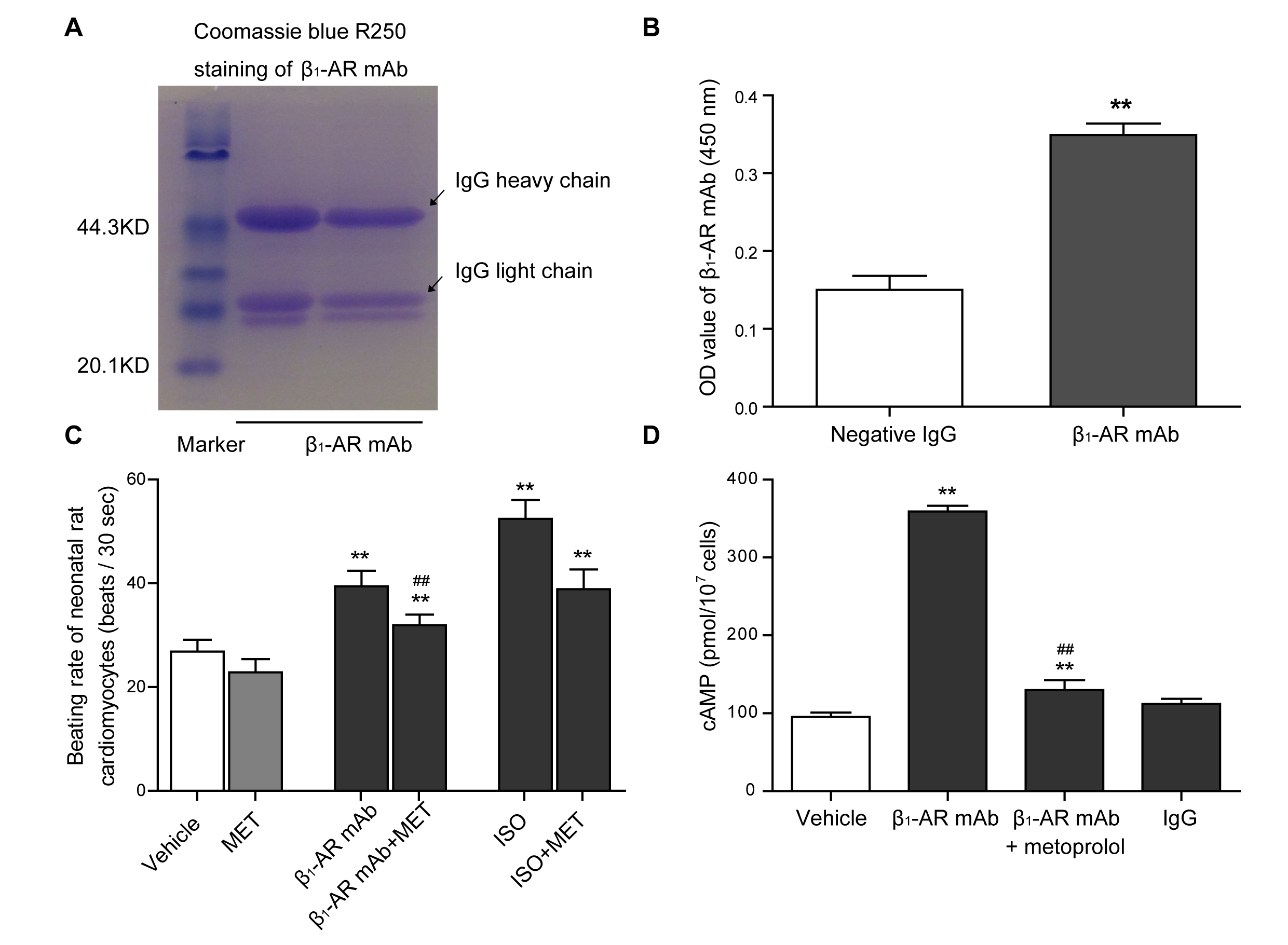
**

**Supplemental Figure 3:** Identification of β_1_-AR mAb from mice ascites purified with the protein G antibody affinity chromatography. (A) Purification of β_1_-AR mAb was determined by the Coomassie Blue R250 staining. The result showed that two straps of 55 KD and 25 KD appeared which represented the heavy chain and the light chain of IgG. (B) Specificity and level of β_1_-AR mAb after purification was detected using SA-ELISA (n = 12 per group). (C) Identification of β_1_-AR mAb (10^-7^ mol/L) activity was performed on neonatal rat cardiomyocytes as evidenced by increased beating rate compared to negative IgG group (n = 16 per group). (D) cAMP levels in supernatants of neonatal rat cardiomyocytes after β_1_-AR mAb stimulation with or without metoprolol for 30 minutes (n = 6 per group). MET metoprolol; ISO isoproterenol. Data are presented as means + SD. (B) ***P* < 0.01 *vs.* negative IgG group; (C-D) ***P* < 0.01 *vs.* vehicle group; *^##^P* < 0.01 *vs.* β_1_-AR mAb group.


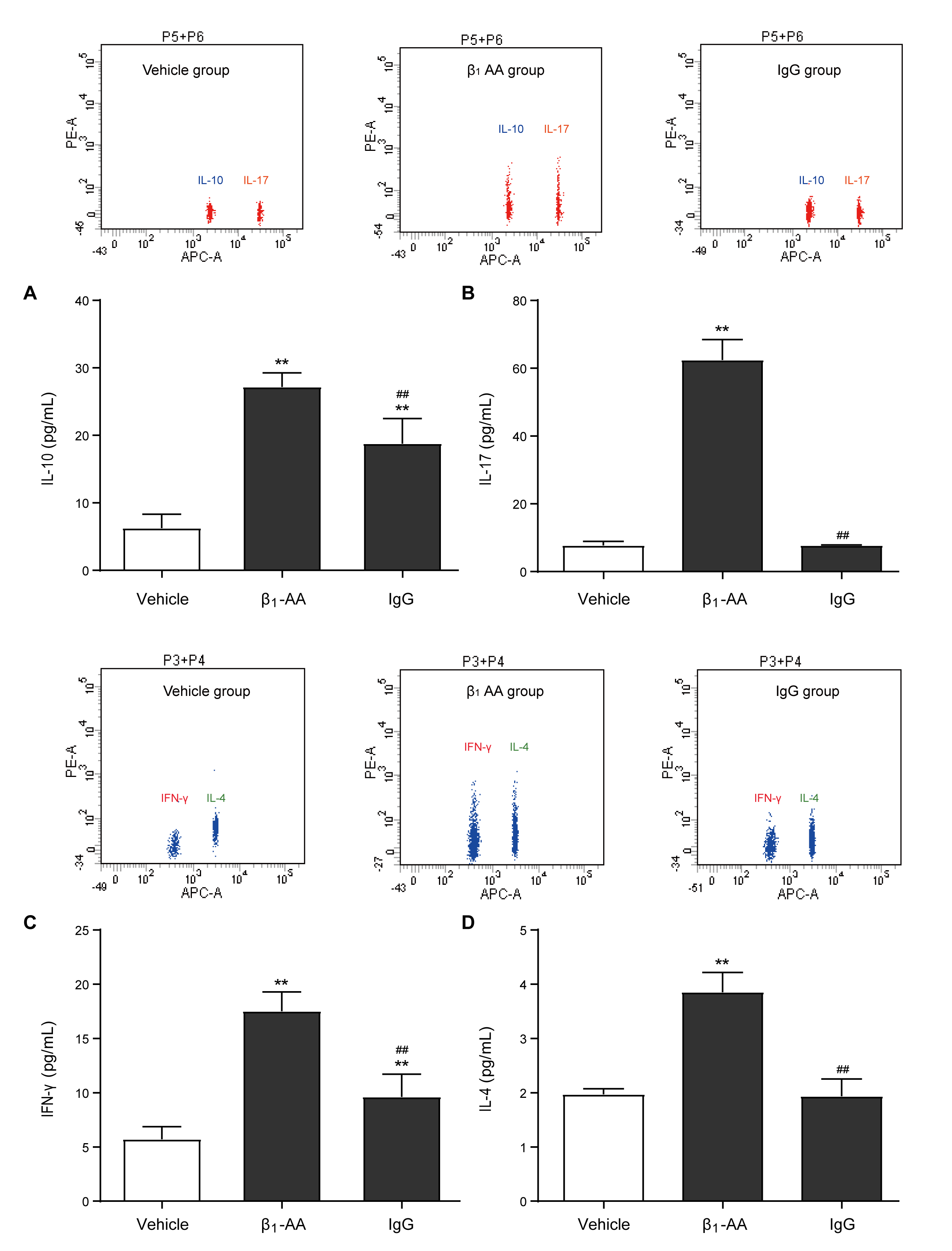


**Supplemental Figure 4:** Concentrations of Treg cytokine (IL-10) (A), Th17 cytokine (IL-17) (B), Th1 cytokine (IFN-γ) (C) and Th2 cytokine (IL-4) (D) in mice sera at the 8^th^ week after β_1_-AA administration were measured by bead-based multi­-analyte flow assay kit (n = 5 per group). Data are presented as means + SD. ***P* < 0.01 *vs.* vehicle group; *^##^P* < 0.05 *vs.* β_1_-AA group.


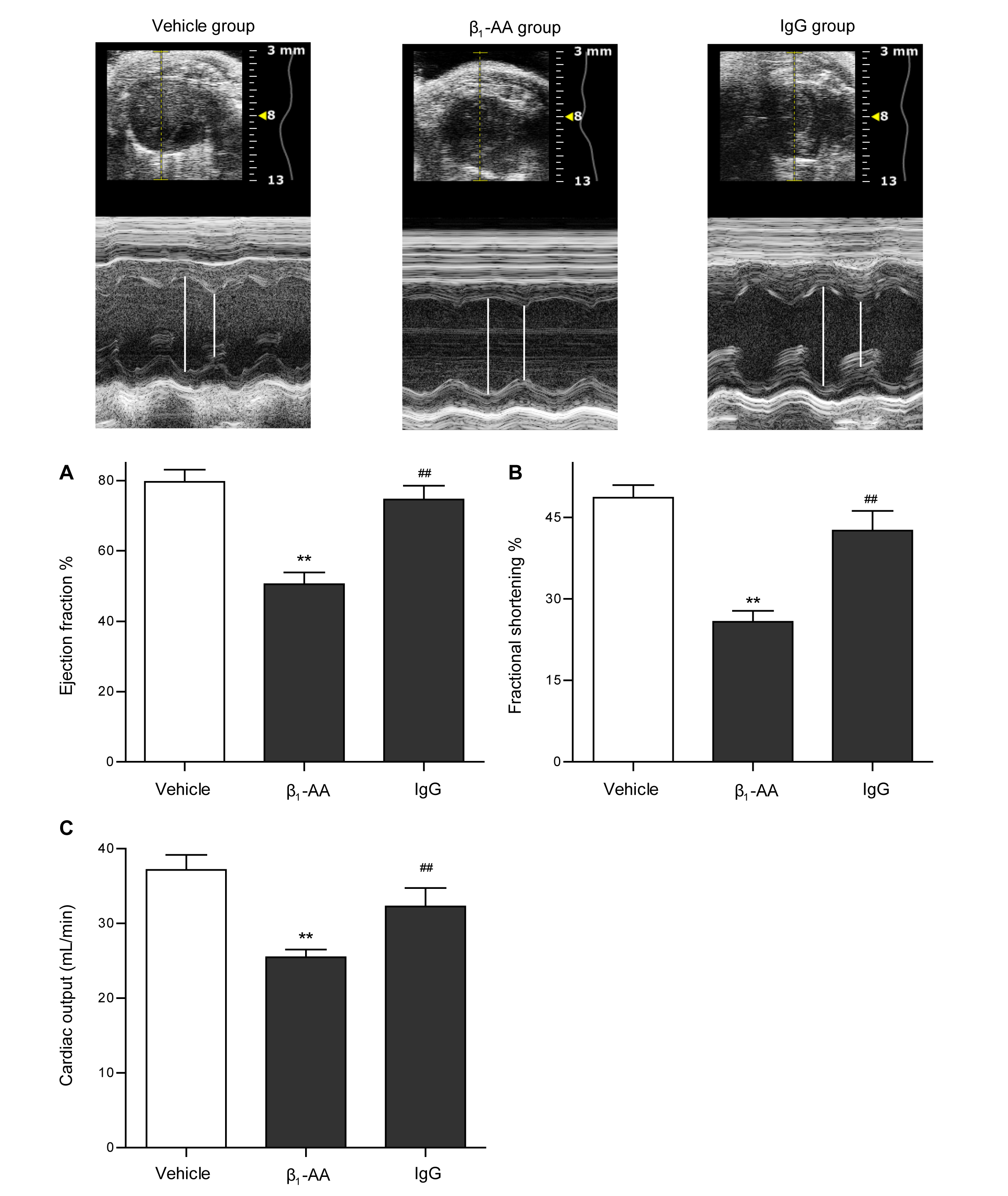


**Supplemental Figure 5:** Changes of cardiac systolic function of β_1_-AA positive mice after eight-week β_1_-AR mAb administration, shown as ejection fractions (A), fractional shortening (B) and cardiac output (C). Images are representative of the echocardiogram of the model mice, and left ventricular end-diastolic or end-systolic diameters are labeled as white stripes. Data are presented as means + SD (n = 5 per group). ***P* < 0.01 *vs.* vehicle group; *^##^P* < 0.05 *vs.* β_1_-AA group.


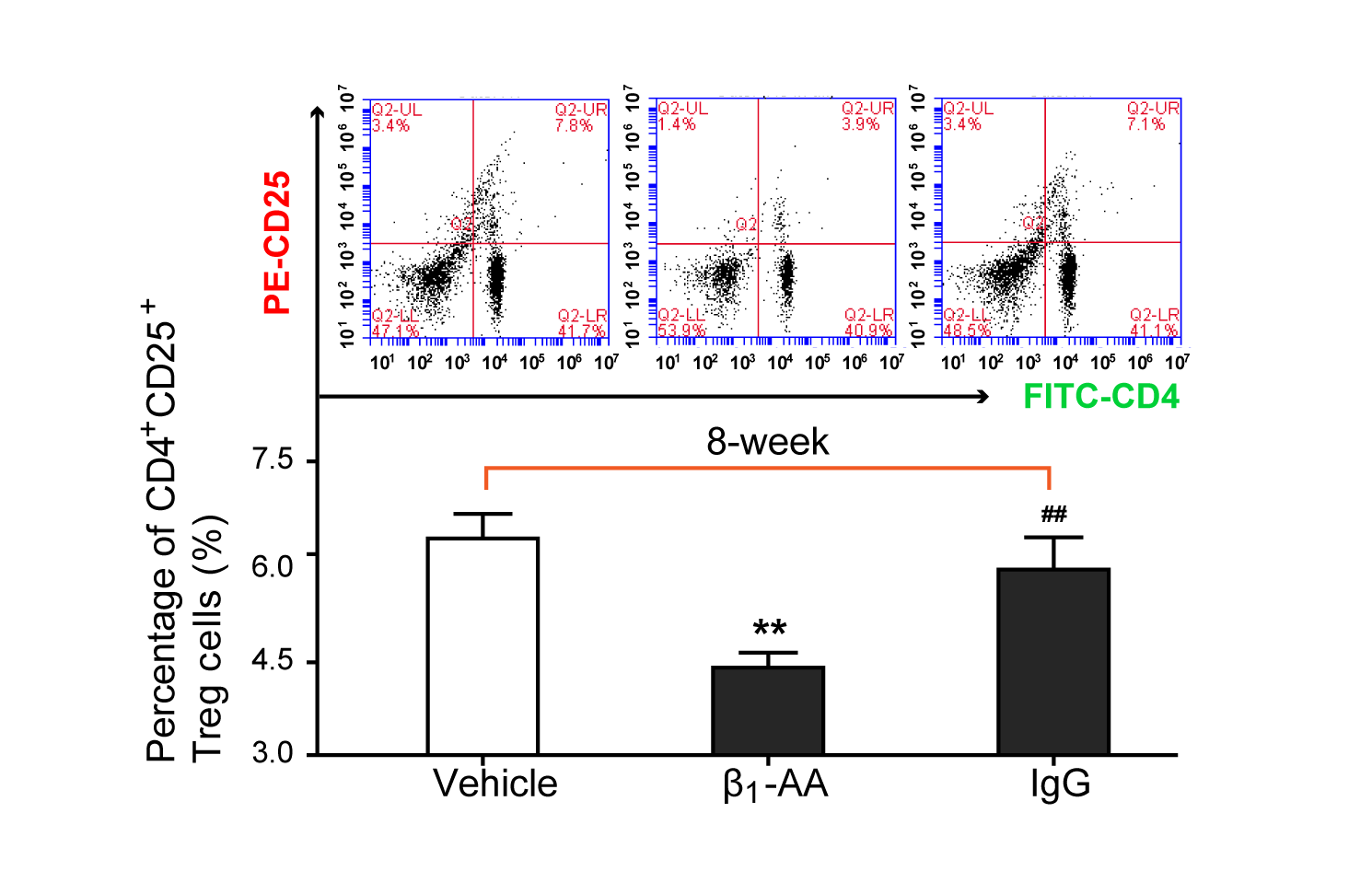


**Supplemental Figure 6:** Frequency of the CD4^+^ CD25^+^ Treg cells in mice peripheral blood at the 8^th^ week after β_1_-AR mAb administration. Data are presented as means + SD (n = 6/per group). ***P* < 0.01 *vs.* vehicle group; *^##^P* < 0.01 *vs.* β_1_-AA group.

**
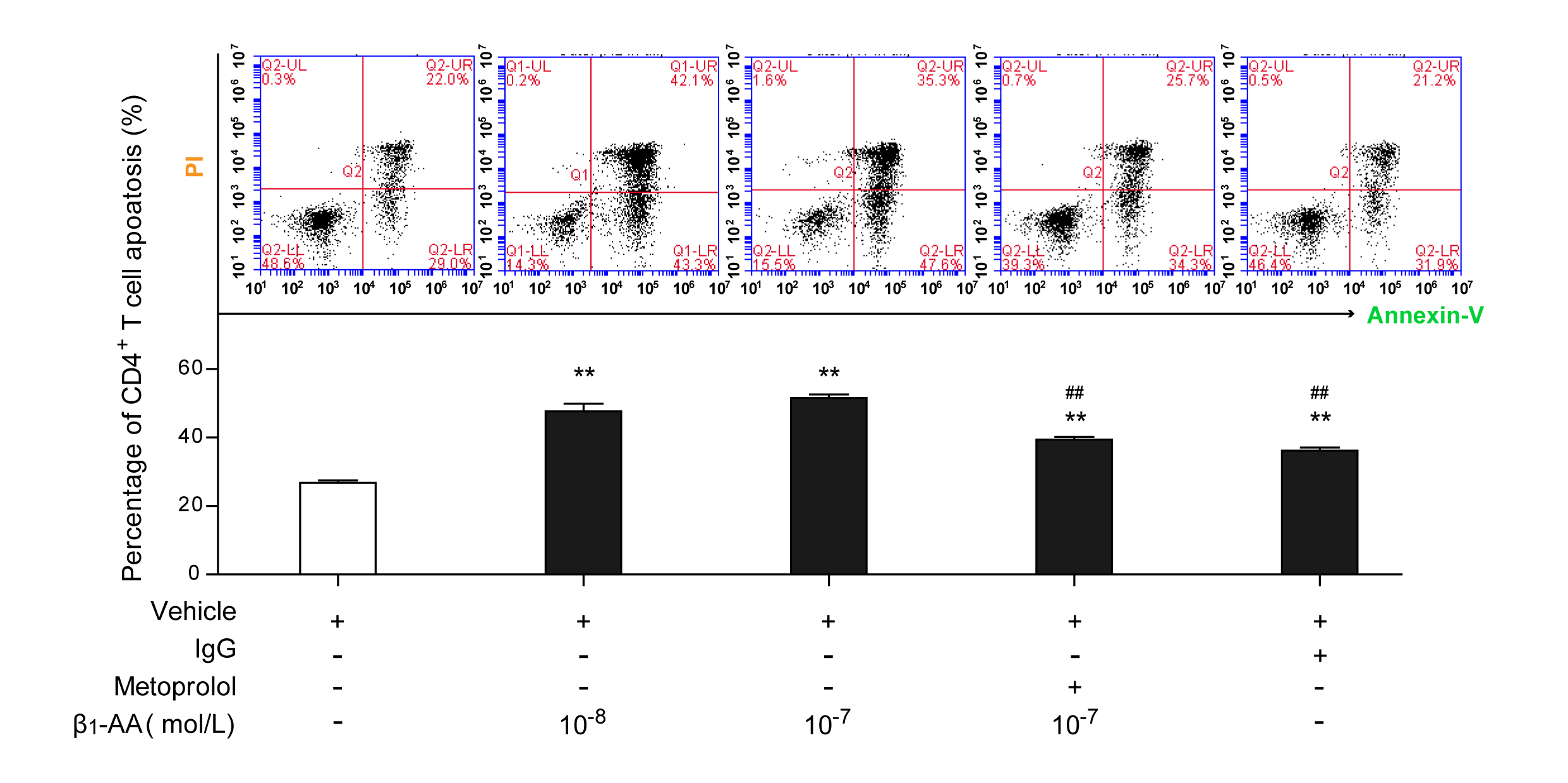
**

**Supplemental Figure 7:** The average percentages of Annexin V-positive CD4^+^ T cells and the mean fluorescence intensity for each population after β_1_-AA administration (10^-8^ and 10^-7^ mol/L) for 6 hours with or without metoprolol. Data are presented as means + SD (n = 6/per group). ***P* < 0.01 *vs.* vehicle group; *^##^P* < 0.05 *vs.* β_1_-AA group.


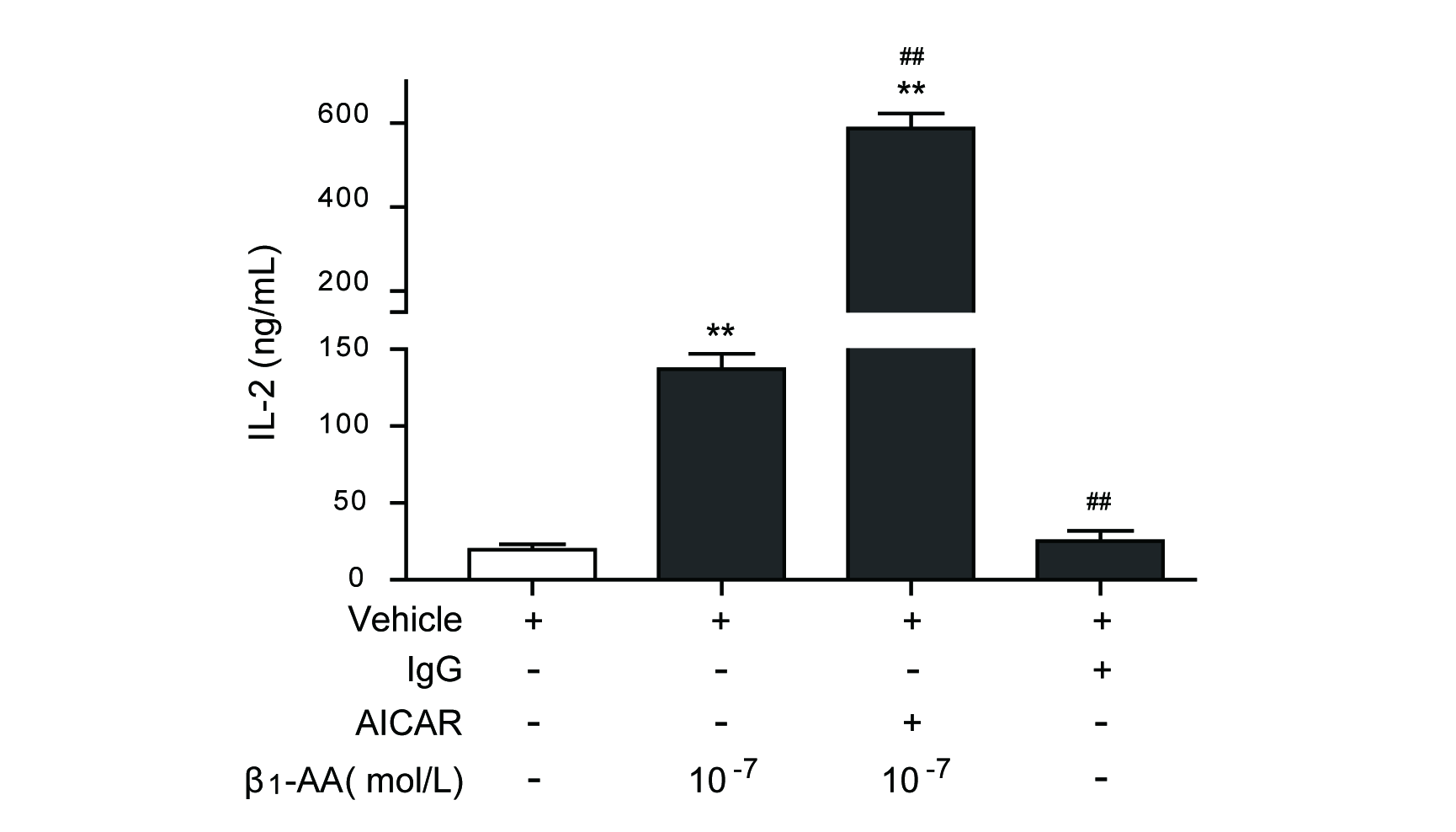


**Supplemental Figure 8:** IL-2 level in the supernatant of activated CD4^+^ T cells after 10^-7^ mol/L β_1_-AA stimulation with or without AICAR for 48 hours measured by radioimmunoassay. Data are presented as means + SD (n = 6 per group). ***P* < 0.01 *vs.* vehicle group; *^##^P* < 0.05 *vs.* β_1_-AA group.

**
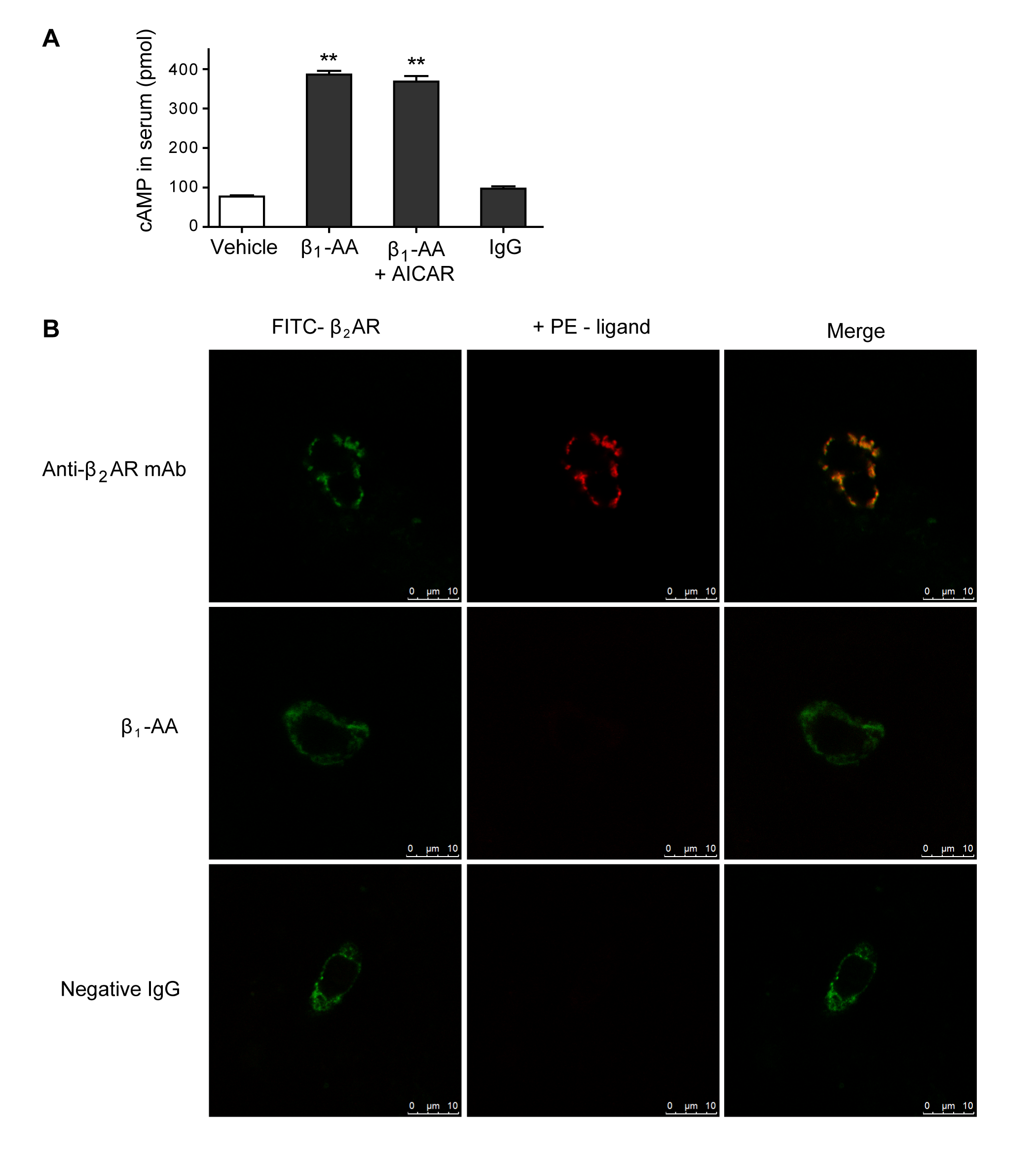
**

**Supplemental Figure 9:** (A) cAMP levels in the supernatants of nTreg cells after thirty-minute β_1_-AA stimulation with or without AICAR (n = 5 per group). Data are presented as means + SD. ***P* < 0.01 *vs.* vehicle group. (B) Visualization of PE-labeled β_1_-AA binding to β_2_-AR on HEK-239 cells. HEK-239 cells were stained with FITC-conjugated second Ab (to anti-β_2_-AR antibody, green) and PE-conjugated second Ab (to ligand, red) and then visualized by confocal microscopy (bars = 50 µm).

**
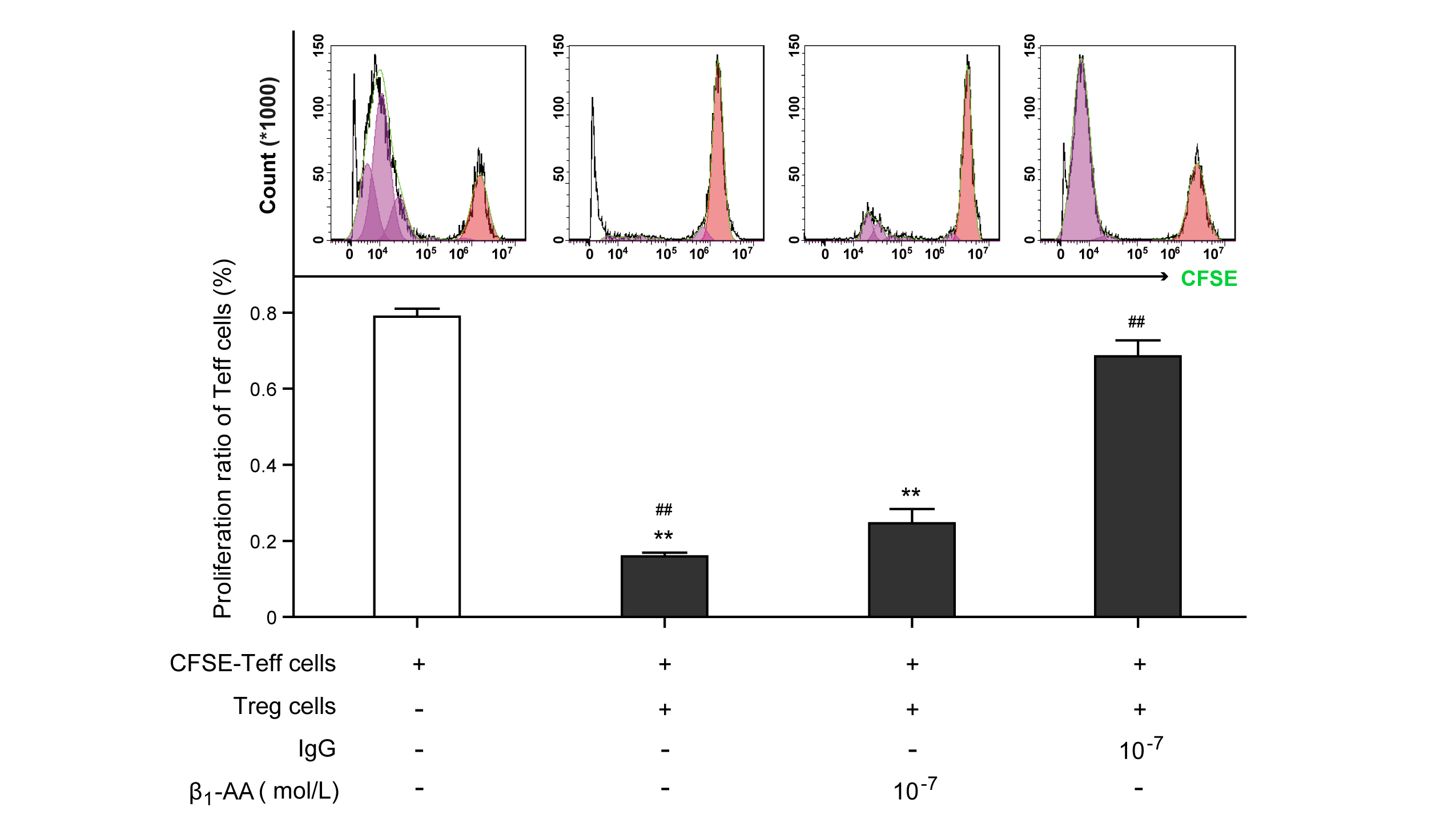
**

**Supplemental Figure 10:** Proliferation assays of CFSE-labeled CD4^+^ CD25^-^ Teff cells cultured alone or with an equal number of unlabeled nTreg cells in the presence of anti-CD3 mAb stimulation (3 μg/mL) for 72 h (proliferation ratio = total count of proliferative/non-proliferating cells) (n = 6 per group). nTreg cells were pre-stimulated with β_1_-AA (10^-7^ mol/L) or with IgG for 48 h. Data are presented as means + SD. ***P* < 0.05 *vs.* Teff cells alone group; *^##^P* < 0.05 *vs.* Teff + β_1_-AA-Treg cells group.

**
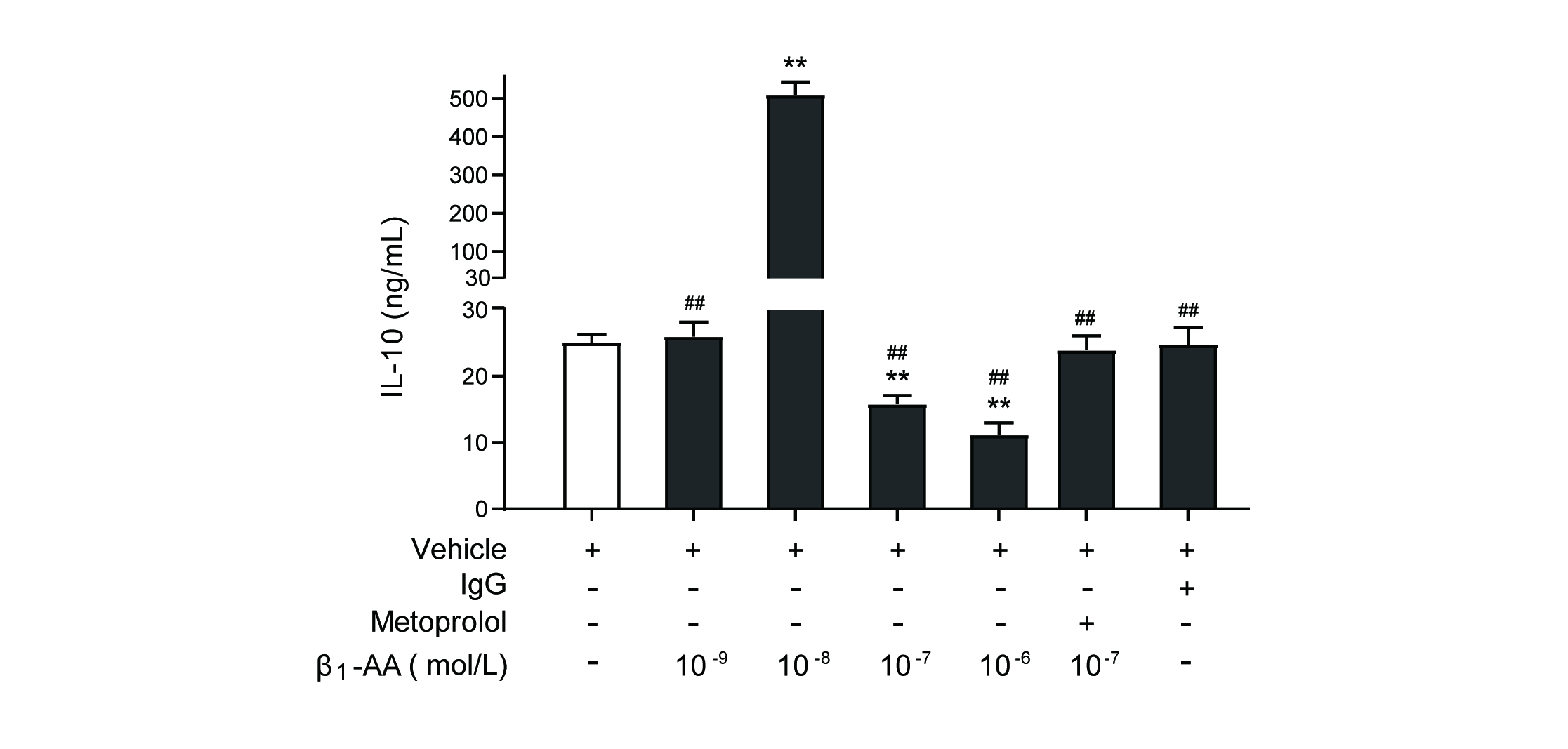
**

**Supplemental Figure 11:** IL-10 level in nTreg cell supernatant after β_1_-AA stimulation (10^-9^, 10^-8^, 10^-7^ or 10^-6^ mol/L) with or without metoprolol for 48 hours measured by radioimmunoassay. Data are presented as means + SD (n = 6 per group). ***P* < 0.01 *vs.* vehicle group; *^##^P* < 0.05 *vs.* 10^-8^ mol/L β_1_-AA group.
